# Extraordinary Mechanical Properties of Composite Silk Through Hereditable Transgenic Silkworm Expressing Recombinant Major Ampullate Spidroin

**DOI:** 10.1038/s41598-018-34150-y

**Published:** 2018-10-29

**Authors:** Zhengying You, Xiaogang Ye, Lupeng Ye, Qiujie Qian, Meiyu Wu, Jia Song, Jiaqian Che, Boxiong Zhong

**Affiliations:** College of Animal Science, Zhejiang University, Hangzhou, 310058 P. R. China

## Abstract

Spider dragline silk is a remarkable material that shows excellent mechanical properties, diverse applications, biocompatibility and biodegradability. Transgenic silkworm technology was used to obtain four types of chimeric silkworm/spider (termed composite) silk fibres, including different lengths of recombinant Major ampullate Spidroin1 (re-MaSp1) or recombinant Major ampullate Spidroin2 (re-MaSp2) from the black widow spider, *Latrodectus hesperus*. The results showed that the overall mechanical properties of composite silk fibres improved as the re-MaSp1 chain length increased, and there were significant linear relationships between the mechanical properties and the re-MaSp1 chain length (p < 0.01). Additionally, a stronger tensile strength was observed for the composite silk fibres that included re-MaSp1, which only contained one type of repetitive motif, (GA)_n_/A_n_, to provide tensile strength, compared with the silk fibres that includedre-MaSp2, which has the same protein chain length as re-MaSp1 but contains multiple types of repetitive motifs, GPGXX and (GA)_n_/A_n_. Therefore, the results indicated that the nature of various repetitive motifs in the primary structure played an important role in imparting excellent mechanical properties to the protein-based silk fibres. A silk protein with a single type of repetitive motif and sufficiently long chains was determined to be an additional indispensable factor. Thus, this study forms a foundation for designing and optimizing the structure of re-silk protein using a heterologous expression system.

## Introduction

The protein-based silk fibres, produced by spiders and silkworms, have fascinated humans for many years due to their excellent mechanical properties; diverse applications in textiles, optics, and biomedicine; and their biocompatibility and biodegradability^1,2^. Spiders can produce up to seven types of silks or glues that are used for various purposes. The diverse uses of spider silks derive from their excellent physical properties, which are tailored for specific purposes, resulting in diverse of mechanical properties.

Dragline silk is of interest primarily because it displays both high tensile strength and extensibility, rendering it tougher than almost all other natural or man-made synthetic materials^3^. The major components of dragline silk are two highly conserved spidroins, MaSp1 and MaSp2. The two spidroins possess two notable structural features: both of them contain a number of repetitive motifs in the primary structure, and both have very long protein chains with a molecular weight (MW) up to 320 kDa^4–6^. According to previous research, the (GA)_n_/A_n_ motifs can impart tensile strength to dragline silk through forming ß-sheets crystalline domain structures, and the GPGXX motif can form ß-turns yielding a spiral structure to impart the elasticity to dragline silk^7^. MaSp1 is primarily composed of (GA)_n_/A_n_ and GGX motifs, while a large proportion of the GPGXX motif is additionally present in MaSp2^8^.

The silk of the best known mulberry silkworm, *Bombyx mori*, is of specific scientific interest because of its industrial-scale production and excellent mechanical properties. The *B*. *mori* silk is wound into a cocoon to protect the pupa during metamorphosis, which requires the silk fibre to be tough rather than strong^9^. In *B*. *mori*, the silk fibre is composed of two proteins: fibroin and sericin. The silk fibroin consists of a heavy (H) chain of 390 kDa^10^ and a light (L) chain of 26 kDa with a disulfide bond, and a glycoprotein called P25 (30 kDa). The H chain also exhibits repetitive motif of (GA)_n_GX, primarily forming ß-sheet structures to form the large crystalline/semicrystalline domains in the silk fibres^11^. In conclusion, spider and silkworm silks are composed of fibroin that typically consists of an iterated repetitive central part flanked by smaller non-repetitive domains; meanwhile, the excellent mechanical properties of silk originate from the unique and highly repetitive sequence in the silk protein, along with its molecular organization and self-assembly at the nanoscale^12,13^, which are occur under physiological and ambient conditions to ensure the structural hierarchy and excellent mechanical properties of the silk fibre^14^.

In the past few years, various heterologous host systems, including bacteria^15,16^, yeast^17^, insects^18,19^, mammalian cell lines^20^, plants^21,22^ and animals^23–25^, were used as platforms to express recombinant spider silk protein for spider-like silk production. Additionally, several attempts have been made to mimic the natural process of producing silk filaments^26^; however, the resulting silk fibres were considerably weaker than native silk. Thus, understanding the relationship between the fibroin protein structure and its mechanical properties is a key step to mimicking the natural silk and to using silk fibre for specific applications.

We speculate that a long protein chain and repetitive motifs in the silk protein could be key factors that are responsible for the extraordinary mechanical properties of silk fibres. Previous studies have indicated that the molecular weight influenced the mechanical properties of polymer silk fibre^27,28^. To investigate the relationship among protein chain length, repetitive motifs and the mechanical properties of dragline silk, the present study generated four types of composite silk fibres using a silkworm silk-gland bioreactor, including different lengths of re-MaSp1 orre-MaSp2 derived from the corresponding dragline silk proteins of *L*. *hesperus*. The results showed that the overall mechanical properties of the composite silk fibres improved with an increasing in the chain length of the recombinant silk protein. This indicated that that the existence of various repetitive motifs in the primary structure and the presence of the same type of repetitive motif and a long protein chain in the silk protein were indispensable important factors for the outstanding mechanical properties of silk fibre. Based on our research results, we speculate that if an artificial spider silk gene expressing proteins with longer length is introduced into the composite silk fibre, then silk fibres with improved mechanical properties would be generated.

## Results

### Transgenic vector design and screening of positive transgenic silkworm lineages

The *MaSp1* gene of *L*. *hesperus* only has a single exon of 9,390 base pairs (bp) encoding 3,129 amino acids (aa) characterized by several highly repetitive units that consist of head-to-tail ligation of four types of primary repetitive sequences, known as Type1, Type2, Type3 and Type4. Each type of primary repetitive sequence contains (GA)_n_ and A_n_ motifs that impart tensile strength to dragline silk by forming ß-sheet structures, and these units are repeated 21 times with near perfect fidelity in the MaSp1 structure^6,29^.

**Figure 1:**
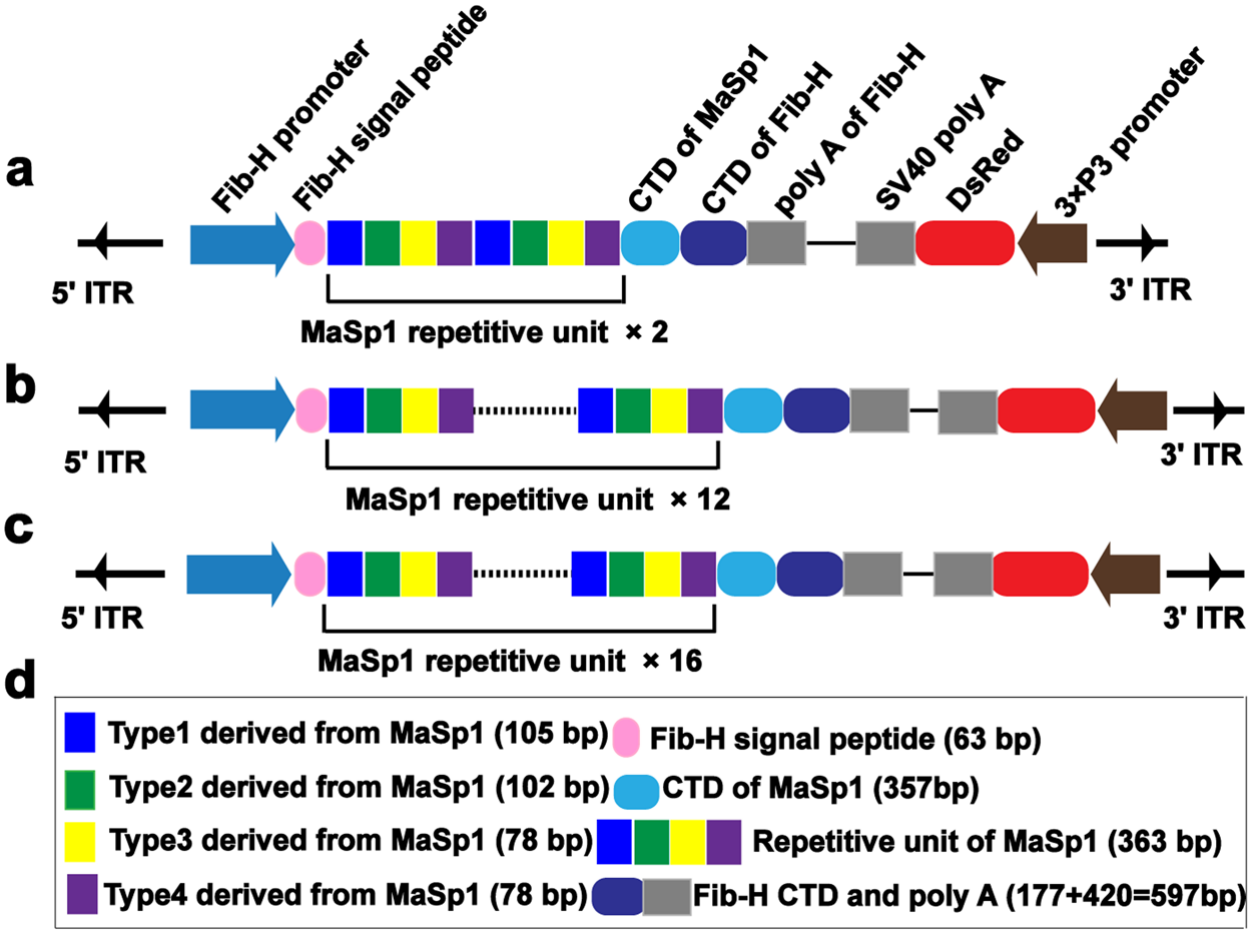
Schematic representation of expression cassettes in the three *piggy*Bac-derived transgenic vectors harbouring re-MaSp1 sequences of different lengths. A schematic of pBac[3 × P3-DsRed]-MaSp1 × 2 (**a**), pBac[3 × P3-DsRed]-MaSp1 × 12 (**b**) and pBac[3 × P3-DsRed]-MaSp1 × 16 (**c**) vectors used to express re-MaSp1, including 2-,12- and 16-fold typical repetitive units of MaSp1, respectively. (**d**) The key elements are shown using boxes with different colours. Fib-H promoter: the primary promoter of *Fib-H* (1269 bp); SP: the signal peptide of *Fib-H*; CTD of MaSp1: C-terminal domain of the *MaSp1*; Fib-HCTD and polyA: the partial C-terminal domain and polyA signal of the *Fib*-*H*; 3 × P3-DsRed was used as the marker gene for screening positive individuals.

In this study, three types of *piggy*Bac-derived vectors containing 2-, 12-, or 16-fold typical repetitive units of *MaSp1* were constructed to specifically express different lengths of re-MaSp1 in the posterior silk glands (PSGs). They were termed pBac[3 × P3-DsRed]-MaSp1 × 2, pBac[3 × P3-DsRed]-MaSp1 × 12 and pBac[3 × P3-DsRed]-MaSp1 × 16 and the expressing proteins had predicted MWs of 39.2 kDa, 133.5 kDa and 171.2 kDa, respectively (Fig. 1, Supplementary Fig. S1a,c and Data S1).

**Figure 2:**
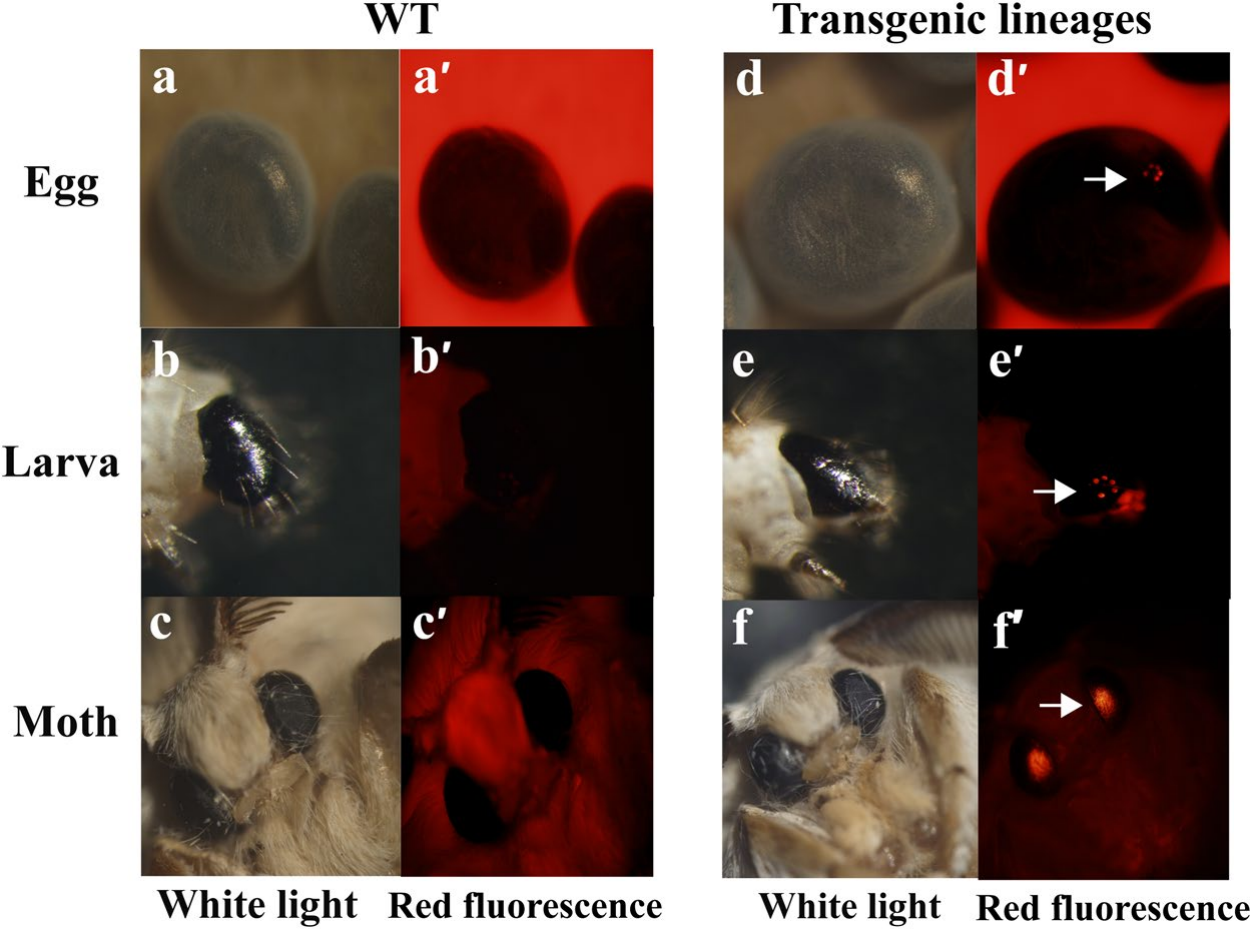
Positive transgenic strains for DsRed-specific expression in the eyes. (**a**,**a’**) G1 egg, (**b**,**b’**) larvae and (**c**,**c’**) moth of the WT strains were viewed under white light and red fluorescence excitation wavelengths, respectively. (**d**,**d’**)G1 egg, (**e**,**e’**) larvae and (**f**,**f’**) moth of the DsRed-positive strains were viewed under white light and red fluorescence excitation wavelengths.

Three types of transgenic silkworm strains that harboured the corresponding *piggy*Bac-derived vector described above were obtained, using the silkworm transgenic technology. These strains were termed MASP1-2, MASP1-12 and MASP1-16 and had 2, 11 and 17 G1 positive transgenic lineages, respectively (Supplementary Table S1). The red-eye phenotype was identified in the eggs, larvae and moths of all positive transgenic lineages (Fig. 2).

**Figure 3:**
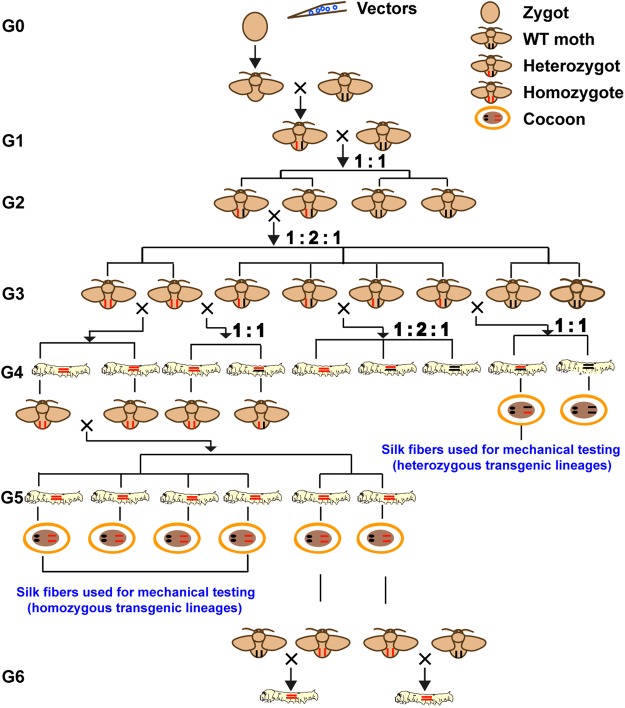
Schematic diagram for generating heterozygous and homozygous transgenic silkworm lineages.

To analyse *re-MaSp1* expression and to investigate the mechanical properties of composite silk fibres from different transgenic lineages, heterozygous and homozygous genotypes of exogenous genes were generated through mating with WT moths or sequential sib-mating until generation4 (G4) and generation5 (G5) for each transgenic lineage (Fig. 3). Precise insertion sites in the *B*. *mori* genome were detected via inverse-PCR. Two, four and four transgenic lineages were randomly chosen as experimental materials for insertion site analysis from the heterozygous transgenic silkworm strains (G4), MASP1-2, MASP1-12 and MASP1-16, respectively. The analysis showed that all 10 transgenic lineages exhibited a single insertion site (Supplementary Fig. S2). In addition, due to the randomness of the integration of exogenous fragments into the TTAA site through the *piggy*Bac transposon-derived vector, except for two pairs of transgenic lineages (MASP1-2-1 and MASP1-2-2, and MASP1-16-8 and MASP1-16-14) that had the same insertion site, the transgenic lineages had different integration sites, even those that harboured the same *piggy*Bac-derived vector.

### Identification of the expression of re-MaSp1 with different sizes in different transgenic silkworm lineages

**Figure 4:**
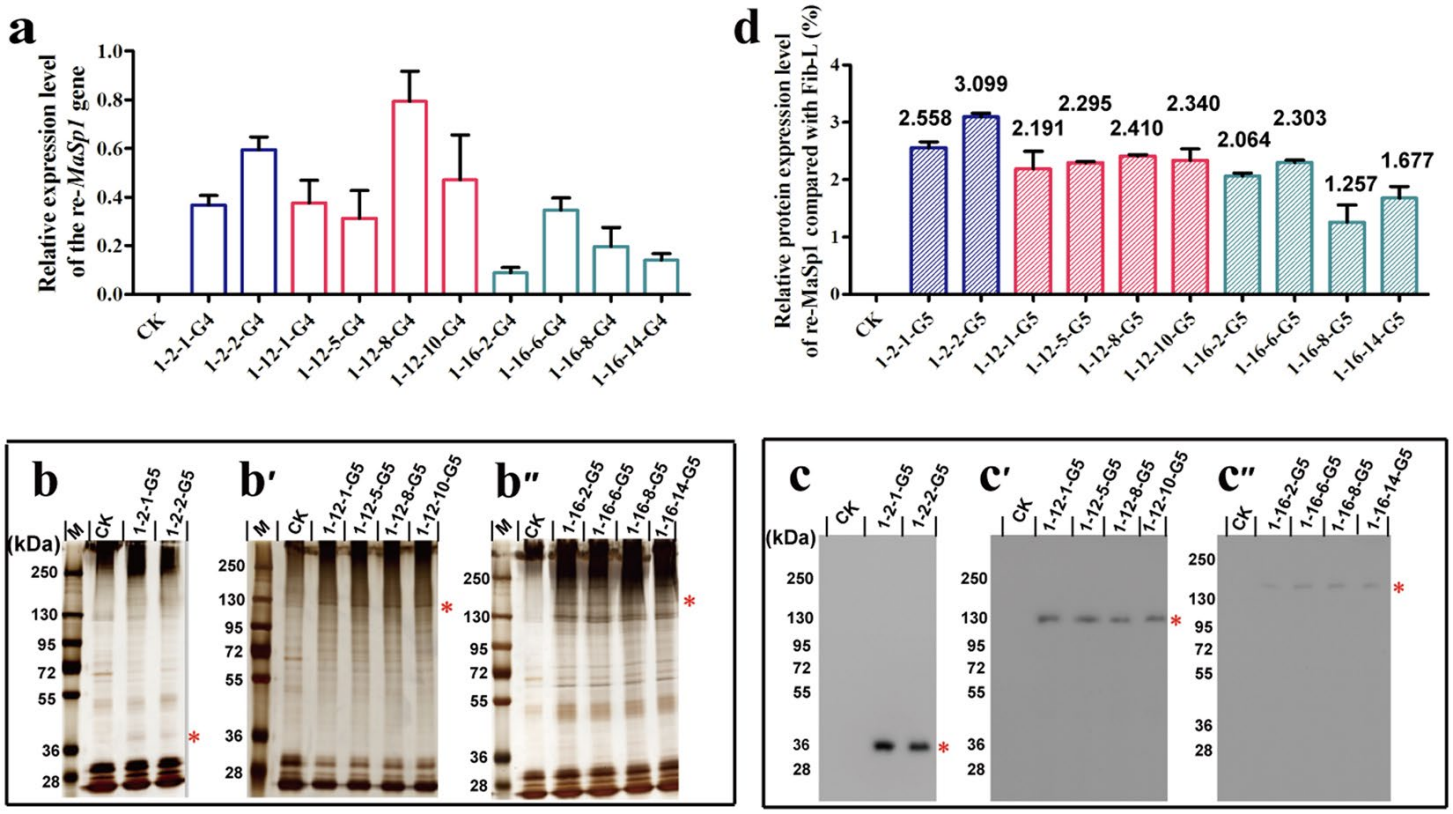
Expression analysis of different lengths of exogenous re-MaSp1. (**a**) The relative expression analysis of different lengths of*re-MaSp1*in the PSGs of the 3^rd^ day of the fifth instar larvae in G4 heterozygous transgenic lineages was performed by qRT-PCR. Mean ± SD values were derived from three independent replicate experiments. Gradient sodium dodecyl sulfate-polyacrylamide gel (5%-12%SDS-PAGE) analysis of the degummed silkworm cocoons was performed, followed by immunoblotting on nitrocellulose membranes; M: 250 kDa protein Marker; CK: the degummed silkworm cocoons of wild-type *Lan10*; (**b**,**c**) the degummed silkworm cocoons of MASP1-2-1 and MASP1-2-2 transgenic lineages; (**b’**,**c’**) the degummed silkworm cocoons of MASP1-12-1, MASP1-12-5, MASP1-12-8 and MASP1-12-10 transgenic lineages; (**b”**,**c”**) the degummed silkworm cocoons of MASP1-16-2, MASP1-16-6, MASP1-16-8 and MASP1-16-14 transgenic lineages. (**d**) Expression analysis of the expression of different lengths of re-MaSp1 relative to the Fib-L protein in homozygous G5 through grey analysis using the Gel-Pro-analyzer4 software. Mean ± SD was derived from three independent replicate experiments.

Quantitative real-time PCR (qRT-PCR) was performed to investigate exogenous *re-MaSp1* expression in the PSGs of the 10 heterozygous transgenic lineages (G4) mentioned above. The expression level of *re-MaSp1* was observed to be different among the transgenic lineages harbouring different transgenic vectors and among those harboured the same type of vector but had different insertion sites. However, the overall trend was that the expression level declined as the number of repetitive units in *re-MaSp1* increased in the transgenic lineages (Fig. 4a).

To further investigate *re-MaSp1* expression at the translational level, degummed silks from the10 homozygous transgenic lineages (G5) described above were analysed via western blotting. The study demonstrated that *re-MaSp1* expressed a single protein band with the predicted size (Fig. 4b-b”,c-c”), which indicated that it was successfully secreted into the transgenic silkworm cocoons and that it formed a composite silk fibre. Figure 4c” presents the degummed silkworm cocoons of 16-fold typical repetitive units of re-MaSp1, which exhibits the highest molecular weight among the exogenous re-MaSp1 in this experiment. Because the expression level of the exogenous gene was also influenced by the molecular weight of the recombinant protein in the transgenic lineages, we speculated that the larger molecular weight of this protein caused its relatively low expression level, resulting in hardly visible and very low bond in the western blot analysis. Briefly, we were able to use the *piggy*Bac system to stably insert the recombinant spider silk gene through multiple generations.

The relative protein level of re-MaSp1 in the composite silk fibre of the homozygous transgenic lineages (G5) was calculated using the fibroin light chain (Fib-L) as a control. The results showed that the re-MaSp1 expression level decreased as the number of amino acids in re-MaSp1 increased (Fig. 4d), indicating that the larger the exogenous gene, the lower the protein expression level, which was consistent with a previous study^16^ and the results of the qRT-PCR study described above.

**Figure 5:**
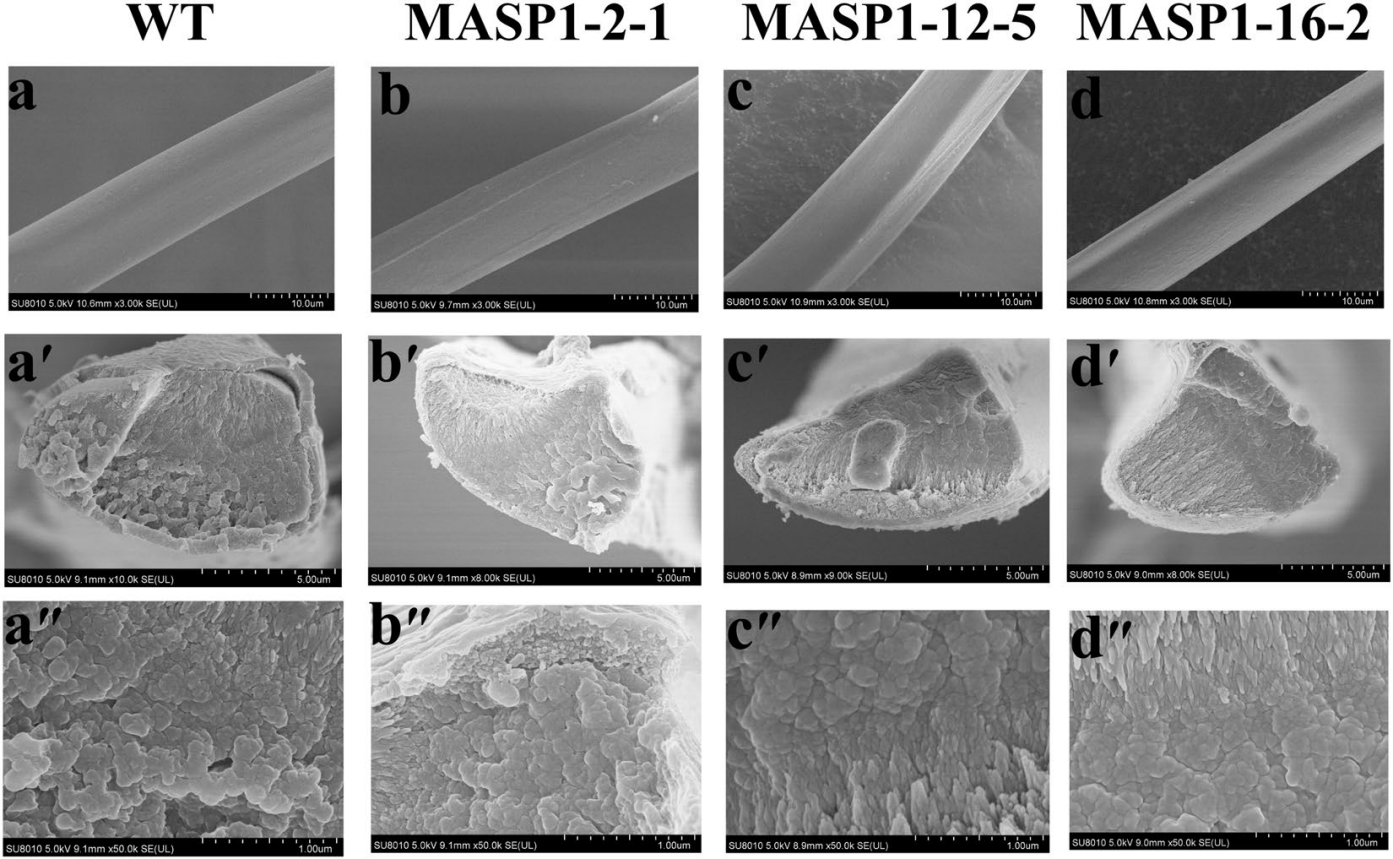
Field emission scanning electron micrographs of the fibres. The surface structure of the silk fibres derived from the non-transgenic silkworm lineages (**a**) and the transgenic silkworm lineages MASP1-2-1 (**b**), MASP1-12-5 (**c**) and MASP1-16-2 (**d**); the cross section structure of the silk fibres derived from the non-transgenic silkworm lineages (**a’**,**a”**) and the transgenic silkworm lineages MASP1-2-1**(b’**,**b”**), MASP1-12-5 (**c’**,**c”**) and MASP1-16-2 (**d’**,**d”**). Scale bars in a-d, 10μm; in a’-d’, 5 μm; in a”-d”, 1 μm.

MASP1-2-1, MASP1-12-5 and MASP1-16-2 were randomly chosen from homozygous transgenic lineages (G5) of MASP1-2, MASP1-12 and MASP1-16, respectively, for scanning electron microscopy analysis of the fibre surface and cross section. The results showed that the morphology structure among WT, MASP1-2, MASP1-12 and MASP1-16 exhibited no obvious differences (Fig. 5).

### Relationship between the re-MaSp1 protein size and the mechanical properties of composite silk fibres

**Figure 6:**
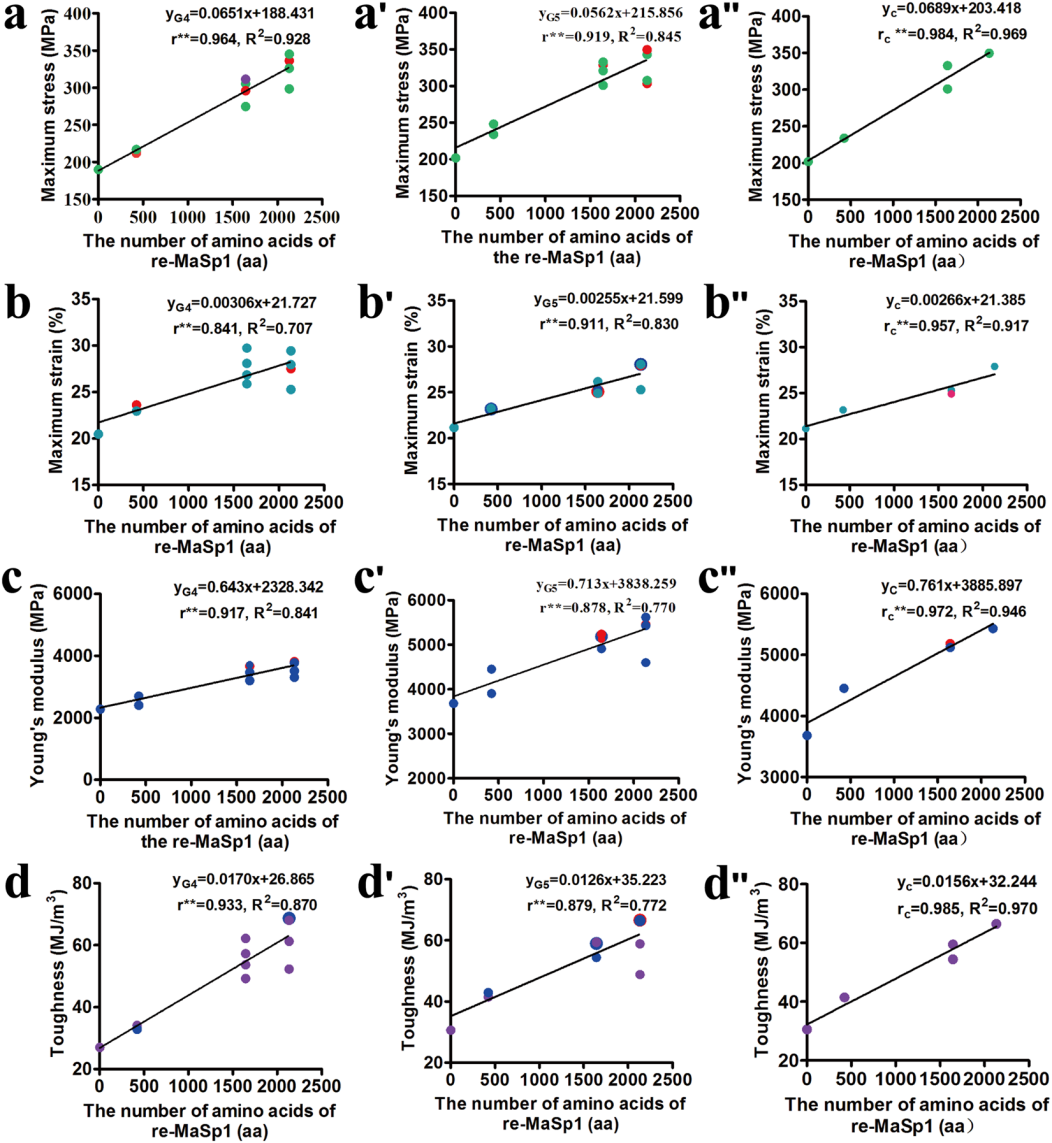
Correlation analysis between the length of re-MaSp1 and mechanical properties of the silk fibres. The correlation analysis between mechanical properties, including maximum stress, maximum strain, Young’s modulus and toughness, and the number of the amino acids (aa) of re-MaSp1, respectively, are shown in (**a**–**d**). (**a**–**d**) The correlation analysis between mechanical properties and the length of re-MaSp1in heterozygous G4; (**a’**–**d’**) the correlation analysis between mechanical properties and the length of re-MaSp1 in homozygous G5; (**a”**–**d”**) the correlation analysis between mechanical properties and the length of re-MaSp1with similar protein levels in homozygous G5. Each dot represents the mean value of the fifteen pieces of silk fibres from each transgenic lineage. Note: Dots with different colours or sizes are used to distinguish close values for different transgenic lineages.

To explore the relationship between the protein chain length and mechanical properties, the mechanical properties of composite silk fibres from 10 heterozygous transgenic lineages (G4) were measured under precisely matched conditions. The results showed that the mechanical properties, including the maximum stress, maximum strain, Young’s modulus and toughness, of the composite silk fibres were different among the transgenic lineages that harboured different vectors, and even among those that harboured the same vector but had different insertion sites; However, the overall trend was that all of the values of the four parameters of the mechanical properties improved with an increase of the number of amino acids in re-MaSp1 (Table 1, Supplementary Table S2 and Fig. S3).Testing the significance of the means indicated significant linear relationships between the re-MaSp1 protein chain length and mechanical properties of the composite silk fibres (Fig. 6a–d).

**Table 1:** Average mechanical properties of the chimeric silkworm/spider silk fibres from the MASP1 transgenic silkworm lineages in heterozygous G4 and homozygous G5.

Transgenic lineages	The number of repetitive unit	Maximum stress (MPa)		Maximum strain (%)		Young’s modulus(MPa)		Toughness (MJ/m^3^)	
		Average	SD	Average	SD	Average	SD	Average	SD
**The average mechanical properties in heterozygous G4**									
Lan10-G4 (n = 15)	Wild type	189.891	44.865	20.482	7.481	2283.280	568.100	27.010	13.947
MASP1-2-G4 (n = 30)	2 × re-MaSp1	214.314	71.643	23.284	5.640	2553.395	771.900	33.545	14.960
MASP1-12-G4 (n = 60)	12 × re-MaSp1	296.839	91.130	27.471	4.079	3514.713	1249.750	55.595	18.576
MASP1-16-G4 (n = 60)	16 × re-MaSp1	326.474	85.535	27.55	4.071	3603.46	1214.625	62.575	19.828
**The average mechanical properties in homozygous G5**									
Lan10-G5 (n = 15)	Wildtype	202.027	52.470	21.149	7.357	3681.163	1019.000	30.573	13.972
MASP1-2-G5 (n = 30)	2 × re-MaSp1	241.043	36.984	23.203	5.268	4178.420	1034.300	41.217	14.596
MASP1-12-G5 (n = 60)	12 × re-MaSp1	320.851	71.681	25.364	3.076	5114.247	1468.750	58.071	15.667
MASP1-16-G5 (n = 60)	16 × re-MaSp1	325.951	61.938	27.318	3.224	5275.978	1466.500	60.199	12.947

Additionally, the mechanical properties of the composite silk fibres from the 10 homozygous transgenic lineages (G5) were measured under the same conditions (Table 1, Supplementary Table S3 and Fig. S4). The data indicated that the homozygous transgenic lineages exhibited reduced variance compared to heterozygous lineages and showed similar results to the heterozygous transgenic lineages (G4). There were significant linear relationships between the re-MaSp1 protein chain length and mechanical properties of the composite silk fibres, and the correlation coefficient of the maximum stress was the highest in both generations, with values of up to 0.964 and 0.919, respectively (Fig. 6a’–d’).

According to the test of significance for the correlation coefficients (r_G4_ and r_G5_) and regression coefficients (b_G4_ and b_G5_) for the same parameters of mechanical properties of composite silk fibres between the heterozygous (G4) and homozygous (G5) transgenic lineages, the results indicated that both correlation and regression coefficients had no significant differences between the heterozygous and homozygous transgenic lineages, and the minimum p value was 0.170 (Table 2). A significant positive correlation relationship was observed between the protein chain length of re-MaSp1 and the mechanical properties of the composite silk fibres, which did not change with changes in the copy number of exogenous genes. Due to the existence of a significant positive correlation relationship between the copy number of genes and the levels of protein expression^30^, the results described above additionally indicated that there was a significant positive correlation relationship that remained unchanged with change in the re-MaSp1 proportion in the composite silk fibre.

**Table 2:** Comparative analysis of the correlation and regression coefficients of the corresponding regression equations established by the protein chain length and mechanical properties of the composite silk fibres between heterozygous G4 and homozygous G5.

	Maximum stress	Maximum strain	Young’ s modulus	Toughness
**Comparative analysis of the correlation coefficient**				
The t value	0.956	−0.726	0.4826	0.721
The p value	0.340	0.470	0.630	0.480
**Comparative analysis of the regression coefficient**				
The t value	0.888	0.672	−0.442	1.401
The p value	0.380	0.510	0.660	0.170

The results described above indicated that the content of exogenous re-MaSp1 in the composite silk fibres declined as the protein chain length increased. To eliminate the influence on the mechanical properties due to the expression level difference of re-MaSp1, we chose to use the mechanical properties of the composite silk fibres from four homozygous transgenic lineages (G5), MASP1-2-1-G5, MASP1-12-5-G5, MASP1-12-10-G5 and MASP1-16-6-G5, which had a similar expression level of re-MaSp1 for further correlation analysis. The results showed that all correctional correlation coefficients (r_c_) of the four parameters were higher than those obtained without eliminating the expression differences, which increased by an average of 0.078, and the smallest value was up to 0.957 (Fig. 6a”–d”). Thus, these results further indicated that there was a linear relationship between the protein chain length and the mechanical properties of the composite silk fibres.

### Comparative analysis of the mechanical properties of the composite silk fibres contributed by re-MaSp1 and re-MaSp2

Similar to *MaSp1*, *L*. *hesperus MaSp2* contains only one enormous exon of 11,340 bp that encodes 3,779 aa with highly repetitive units. These repetitive unit are organized into four types of primary repetitive sequences, Type1′, Type2′, Type3′ and Type4′, and all of which include both the (GA)_n_ and the A_n_ motifs as well as the GPGXX motif. The (GA)_n_ and the A_n_ motifs provide the tensile strength of the silk fibre, the GPGXX motif provides elasticity through formation of ß-spiral spring structures^29^. Nevertheless, the structural component of the repetitive unit of MaSp2, which primarily consists of sequential Type1′-Type1′-Type4′, is different from that of MaSp1, and there are 22-fold repetitive units with little differences in the sequence and length throughout the protein structure^6^.

To compare the mechanical properties of the silk fibres contributed by MaSp1 and MaSp2, the *piggy*Bac-derived vector, pBac[3 × P3-DsRed]-MaSp2 × 24, which contains 24-fold typical repetitive units of *MaSp2* from *L*. *hesperus* was constructed to express re-MaSp2, which contained1,926 aa and had a predicted MW of approximately 160 kDa (Supplementary Figs S1b,c, S5 and data S2).

The transgenic strain harbouring the pBac[3 × P3-DsRed]-MaSp2 × 24 vector was obtained using silkworm transgenic technology, was termed MASP2-24, and had 6 G1 positive transgenic lineages (Supplementary Table S1). The G4 heterozygous and G5 homozygous genotypes of*re-MaSp2* were generated through the method described above. Four transgenic lineages were randomly chosen for further analysis. The results showed that exogenous *re-MaSp2* was integrated into the genome of *B*. *mori* with a single insertion site (Supplementary Fig. S2), stably expressed, and inherited in the transgenic lineages (Supplementary Fig. S6), which indicated that re-MaSp2 was successfully secreted into the transgenic silkworm cocoons, forming composite silk fibres. The results of the scanning electron microscopy analysis showed that the morphologic structures between the transgenic lineage and WT exhibited no obvious differences (Supplementary Fig. S7).

The mechanical properties of the composite silk fibres from the heterozygous G4 and homozygous transgenic lineages (G5) were measured under precisely matched conditions (Table 3, Supplementary Tables S2 and S3). MASP2-24-4-G5, which exhibited a similar protein expression level to re-MaSp2 compared with re-MaSp1 according to correctional correlation coefficients (r_c_) analysis, was used for further analysis. The amino acid number of re-MaSp2, 1926, was entered into regression equations, y_c_ (Fig. 6a”–d”), which were established by the data from the MASP1-G5 transgenic lineages with similar expression levels, to obtain the calculated values and confidence intervals of the maximum stress and maximum strain parameters (Table 4). The comparative analysis showed that the experimental testing value of the maximum stress on behalf of the tensile strength of silk fibres from the transgenic lineages was lower than the calculated value evaluated by the regression equations, y_c_, and that the value was significant. In addition, the experimental testing value of the maximum strain on behalf of the elasticity of the fibres exhibited no obvious difference compared with the calculated values.

**Table 3:** Average mechanical properties of the chimeric silkworm/spider silk fibres from the MASP2 transgenic silkworm lineages in heterozygous G4 and homozygous G5.

Transgenic lineages	Number of repetitive units	Maximum stress (MPa)		Maximum strain (%)		Young′s modulus (MPa)		Toughness (MJ/m^3^)	
		Average	SD	Average	SD	Average	SD	Average	SD
**The average mechanical properties in heterozygous G4**									
Lan10-G4 (n = 15)	Wild type	189.891	44.865	20.482	7.481	2283.280	568.100	27.010	13.947
MASP2-24-G4 (n = 60)	24 × re-MaSp2	302.488	66.475	26.075	3.857	3255.005	1242.250	52.858	14.735
**The average mechanical properties in homozygous G5**									
Lan10-G5 (n = 15)	Wild type	202.027	52.470	21.149	7.357	3681.160	1019.000	30.573	13.972
MASP2-24-G5 (n = 60)	24 × re-MaSp2	313.880	74.628	26.662	4.620	5184.918	1397.500	58.474	18.921

**Table 4:** Mechanical properties of composite silk fibres that contained re-MaSp2 in G5 was estimated based on the linear equation, y_c_, and adjusted by the similar expression level of re-MaSp1 in homozygous G5.

	Calculated values	Experimental values (the difference between the experiment and calculated values) MASP2-24-4-G5	Lower limit (p = 0.05)	Upper limit (p = 0.05)
Maximum stress (MPa)	336.119	303.67(−32.45)*	310.79	361.45
Maximum strain (%)	26.508	25.36(−1.15)	24.87	28.14

^*^Notes: Values marked with mean significant difference.

## Discussion

A widely accepted model of the silk fibre structure is that it is a semicrystalline biopolymer and consists of many nanofibrils, among which the ß-sheet nanofibrils are composed of the crystalline domains and the amorphous domains are in a well-organized manner^13^. Comparative analysis of the mechanical properties of composite silk fibres, including re-MaSp1 and re-MaSp2, indicated that the tensile strength of the composite silk fibre that contained re-MaSp1 was higher than that for the fibre that contained re-MaSp2. This was because re-MaSp1 included up to 46.28% (GA)_n_ and A_n_ motifs (52/121 = 46.28%), which provided tensile strength to the silk fibre, whereas the GPGXX motif for elasticity was introduced in re-MaSp2 with the same protein chain length as re-MaSp1, resulting in a decrease in the proportion of (GA)_n_ and A_n_ motifs to 41.66% (30/72 = 41.66%). However, although the GPGXX motif was introduced into re-MaSp2, it was not similar to the flagelliform silk protein (Flag), which contained at least 43 continuous GPGXX motifs^31^. Therefore, the composite silk fibres containing re-MaSp2 exhibited slightly improved elasticity; however, this improvement did not reach a significant level. These results demonstrated that the silk protein that included many and single functional motifs could improve the corresponding mechanical properties of the silk fibre.

Additionally, based on the comparative analysis of the composite silk fibres that included different lengths of re-MaSp1, extremely significant linear relationships were observed between the protein chain length and mechanical properties of the composite silk fibres. Furthermore, the mechanical properties improved as the protein chain length increased. Varying the chain length of recombinant spider silk in silkworms has never been reported. In addition, the scanning electron microscopy analysis demonstrated that there were no obvious differences in the morphological structures of the silk fibres between the transgenic lineages and WT strains, which suggested that the significant improvement in the mechanical properties was primarily because of the increase in the protein chain length and that there was no obvious relationship with the morphology.

Thus, we speculated that the various repetitive motifs were the primary molecular basis for the unique mechanical properties of various spider silks, while proteins with a long molecular chain could be important for the outstanding mechanical properties because longer proteins encompass more repetitive motifs than shorter proteins to enhance their functions. In other words, to achieve high-performance protein-based silk fibres, the fibrous proteins need to possess not only unique repetitive motifs but also sufficiently long protein chains to ensure adequate iterations of these repetitive motifs, which indicates that a protein with several single functional motifs and sufficiently long protein chains is a vital prerequisite for the outstanding mechanical properties of spider silk fibres.

In nature, there are several instances of silk fibres that possess unique mechanical properties because the protein chain contains several repetitive motifs. Flagelliform silk primarily consists of Flag and has a MW of up to approximately 500 kDa, which contains the GPGXX motif with at least 43 more continuous repeats than MaSp2^31^. Therefore, flagelliform silk possesses the highest elasticity among the various spider silks at up to 200%, and it exhibits an approximately 10-fold extensibility compared with dragline silk^32^. Conversely, minor ampullate silk is primarily composed of minor ampullate spidroin1 (MiSp1) and minor ampullate spidroin2 (MiSp2). Both MiSp1 and MiSp2 possess (GA)_n_/(A)_n_ motifs similar to those in MaSp1; however, MiSp1 and MiSp2 have lower MWs, especially MiSp2, which is only 70% of MaSp1^33^. Thus, it exhibits approximately 40% tensile strength compared with dragline silk^29^.

Silkworm cocoon silk is composed of approximately 75% insoluble fibrous proteins that primarily include two proteins: fibroin heavy chain (Fib-H) and fibroin light chain (Fib-L) at a 1:1 molar ratio^34^. Fib-H has a high MW of up to 390 kDa, which is similar to that for the dragline silk protein, and is organized into highly repetitive motifs to form a ß-sheet structure, which is believed to be the molecular basis for the tensile strength of silkworm silk^10^. These instances are further evidence for the hypothesis proposed in our study.

Among different species of spiders, even between spiders and silkworms belonging to different classes and with a great distance between their taxonomic statuses, the major constituent proteins of their silk fibres are long-chain proteins with highly repetitive sequences, which indicate that this type of large molecular structure is perfect for unique mechanical properties. These insights further indicated that adopting long-chain proteins with highly repetitive structures into protein-based natural silk fibres with outstanding mechanical properties could be an inevitable result of biological evolution.

The mechanical properties of silk fibres composed of longer chain proteins are better than those of fibres composed of smaller chain proteins. One underlying reason for this difference could be that proteins contain various amino acids that are linked together by peptide bonds, and thus, smaller proteins depend on intermolecular forces, such as van der Waals forces, more than the longer proteins to extend their molecular chain to the same length. Silk fibres that depend on strong peptide bonds could generate more stable structures than fibres based on weak Van der Waals forces^16^.

In a previous study, recombinant spider silk proteins with various numbers of repetitive units derived from *N*. *clavipes* MaSp1 were expressed in *E*. *coli*, and the mechanical properties of spun fibres were related to the protein length^16,35^. Consistently, our results also suggested that there was a relationship between the protein length and mechanical properties, which indicates that the results based on our study are reliable. These recombinant silk proteins expressed in *E*. *coli* were modified by methionine and were spun into fibres using a man-made spinning process^16^. However, spider silk fibres are produced in a highly sophisticated hierarchical spinning process that includes a high protein concentration, pH and ion gradients, as well as shear forces, which are crucial for forming the structure and the outstanding mechanical properties of the silk fibres^36–38^. This process cannot be matched by the man-made spinning processes developed so far^39^. Thus, the mechanical properties are based on the underlying silk protein structure, their self-assembly and the spinning process. Considering the fibre spinning process, a series of *piggy*Bac-derived vectors that contained different lengths of*re-MaSp1* with highly similar sequences to the native *MaSp1 *of *L*. *hesperus* were used to obtain various composite silkworm/spider silk fibres using *B*. *mori* as a platform, which has a similar mechanism for the spinning process to that of silk formation in the spider^40^. The amino-acid composition is highly similar between the silkworm and spider silk proteins, which consist of glycine-rich and alanine-rich proteins^10,41^. Therefore, the mechanical properties of the silk fibres described above would be similar to those of the natural silk fibres used in this study.

If natural spider silks could be used in this study, the relationship between the protein chain length and the mechanical properties of dragline silk from *L*. *hesperus* would be more accurately presented; however, to the best of our knowledge, natural spider silks composed of different protein lengths were not obtained in previous studies. Thus, our analyses used composite silk fibres, including different lengths of re-MaSp1 with the corresponding numbers of repetitive unit sequences and used *B*. *mori* silk as the reference. This approach could provide a good alternative approach for producing silk. The results obtained in our study would be credible because the background of *B*. *mori* silk exhibited no obvious interference with the mechanical properties of composite silk fibres, including different lengths of re-MaSp1.

Currently, various heterologous host systems, including bacteria^15,16^, yeast^17^, insects^18,19^, mammalian cell lines^20^, plants^21,22^ and animals^23–25^, have been used as platforms to express recombinant spider silk protein. Based on our research, we speculate that if artificial spider silk genes expressing proteins with longer lengths than the natural spider gene were introduced into the silkworm silk fibre, then silk fibres with superior mechanical properties, including stress, strain, Young’s modulus, and toughness, would be generated.

Moreover, advanced genetic manipulation technologies, including TALENs (transcription activator-like effector nucleases) and CRISPR/Cas9 (clustered regularly interspaced palindromic repeats/CRISPR)^42–44^, have enabled efficient genome editing experiments in *B*. *mori*^45–49^. If endogenous silk genes or theregulatory factors inthe silkworm transgenic lineages obtained in our study were mutated by employing thesegenome editing technologies to reduce the silk protein expression levels, then the expression of the exogenous recombinant spider silk proteins would be enhanced. Silk fibres from the silkworm transgenic lineages would be further improved, enabling potentially broad applications of the silkworm silk fibre.

## Conclusion

The present study indicated four important conclusions: (1) The recombinant spider silk gene stably inserted through multiple generations was obtained using the *piggy*Bac system; (2) the mechanical properties for homozygous transgenic lineages exhibited reduced variance in comparison to heterozygous transgenic lineages, and both correlation and regression coefficients exhibited no significant differences between the heterozygous and homozygous transgenic lineages; (3) the overall mechanical properties of the composite silk fibres improved as the re-MaSp1 chain length increased and exhibited extremely significant linear relationships with the protein chain length; and (4) a protein with several single functional motifs and sufficiently long protein chains is a vital prerequisite for the outstanding mechanical properties of silk fibres.

## Methods

### Vector Design and Construction

**Figure 7:**
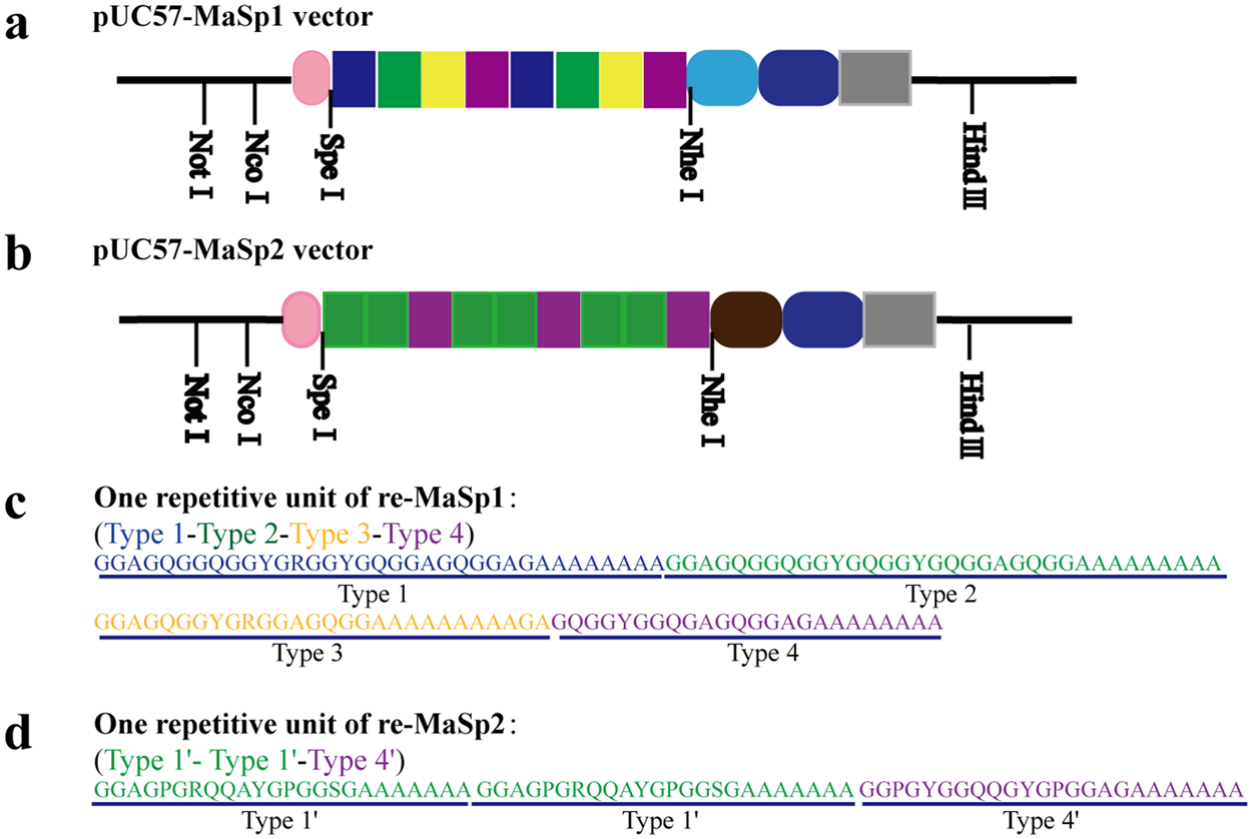
Schematic representation of synthetic spider silk gene and structural motifs. (**a**) The pUC57-MaSp1 vector including two repetitive units of re-MaSp1; (**b**) the pUC57-MaSp2 vector including three repetitive units of re-MaSp2; (**c**) the detailed amino acid sequence of one repetitive unit of re-MaSp1, Type1, Type2, Type3 and Type4 are the structural units derived from the black widow spider, *L*. *hesperus*; (**d**) the detailed amino acid sequence of one repetitive unit of re-MaSp1, Type1′ and 4′ are the structural units derived from the MaSp2 protein of the black widow spider, *L*. *hesperus*.

A series of *piggy*Bac vectors containing 2, 12 or 16 times the number of typical repetitive units in MaSp1 or 24 times the number of typical repetitive units of MaSp2 derived from the black widow spider, *L*. *hesperus*^6^, were designed. The codon of the typical repetitive unit sequences of *MaSp1* and *MaSp2* were optimized according to codon usage in *B*. *mori*^10^ to ensure a high level of expression of re-MaSp1 and re-MaSp2 in *B*. *mori*. The sequence of the signal peptide of silkworm *Fib*-*H*, two sequential typical repetitive units of *MaSp1* or three sequential typical repetitive units of *MaSp2*, the C-terminal domain (CTD) of the *MaSp1* or *MaSp2*, and the partial C-terminal domain (CTD), as well as the polyA signal of the *Fib*-*H* gene were synthesized (GENEWIZ, China) and then sub-cloned into the pUC57 vector to generate intermediate pUC57-MaSp1 or pUC57-MaSp2 vectors, which carried *Nco*І, *Spe*І, *Nhe*І and *Hind*III restriction sites (Fig. 7, Supplementary Fig. S1a,b).

Then, a series of *piggy*Bac-derived vectors were generated using the doubling strategy^7^. Briefly, by simultaneously performing two double digestion reactions (*Spe*І-*Hind*III and *Nhe*І-*Hind*III), two fragments containing 2-fold typical repetitive units of *MaSp1* were obtained. The two fragments were ligated together, which led to a doubling of the length of the typical repetitive units of *MaSp* using T_4_ ligase (TaKaRa, China). After several rounds of cloning, intermediate vectors containing 2-, 4-, 8-, 12-, and 16-fold typical repetitive units of *MaSp1* were obtained, which were designated pUC-MaSp1 × 2, pUC-MaSp1 × 4, pUC-MaSp1 × 8, pUC-MaSp1 × 12 and pUC-MaSp1 × 16, respectively. In parallel, intermediate vectors containing 3-, 6-, 12-, 18-, and 24-fold typical repetitive units of *MaSp2* were also obtained, which were designated pUC-MaSp2 × 3, pUC-MaSp2 × 6, pUC-MaSp2 × 12, pUC-MaSp2 × 18 and pUC-MaSp2 × 24, respectively.

The primary promoter of the *Fib*-*H* gene (pFib-H) was produced by PCR amplification with genomic DNA isolated from the silk glands of *B*. *mori Lan10* and sub-cloned into the pMD19-T vector (TaKaRa, China) to create the intermediate vector pMD19-T-pFib-H. The gene-specific primers (pFib-H-F/R) with *Xho*І at the 5′- and *Nco*І at the 3′- end are shown in Supplementary Table S4. The fragments of the *Fib*-*H* gene primary promoter from the intermediate vector pMD19-T-pFib-H were digested with *Xho*І and *Nco*І and then inserted into the corresponding sites of the pBac6212 basic transgenic vector (conserved in our lab). This step resulted in a vector that was designated pBac-pFib-H.

The fragments of key modules, including 2-, 12-, or 16-fold typical repetitive units of *re*-*MaSp1* and 24-fold typical repetitive units of *re*-*MaSp2* were chosen and excised from the pUC-MaSp1 × 2, pUC-MaSp1 × 12, pUC-MaSp1 × 16, or pUC-MaSp2 × 24 vectors described above with *Nco*І and *Mun*І, and then the sequences were sub-cloned into the corresponding downstream sites of pFib-H in the intermediate vector pBac-pFib-H. This step yielded four vectors named pBac-MaSp1 × 2, pBac-MaSp1 × 12, pBac-MaSp1 × 16 and pBac-MaSp2 × 24.

Finally, the fragments of 3 × P3-DsRed-SV40 were cut with *Bgl* II and *Afl* II from the pBac7785 basic transgenic vector (which was constructed and maintained in our lab), and then sub-cloned into the corresponding sites of pBac-MaSp1 × 2, pBac-MaSp1 × 12, pBac-MaSp1 × 16 and pBac-MaSp2 × 24 described above. This final step obtained four separate *piggyBac*-derived vectors named pBac[3 × P3-DsRed]-MaSp1 × 2, pBac[3 × P3-DsRed]-MaSp1 × 12, pBac[3 × P3-DsRed]-MaSp1 × 16 and pBac[3 × P3-DsRed]- MaSp2 × 24 for additional germline transformation experiments.

DNA manipulations were carried out according to standard protocols, i.e., PCR amplified, gel-purified, ligated, and sub-cloned. The vectors containing cloned fragments were confirmed by DNA sequencing or restriction enzyme analysis.

### Silkworm Strains and Germ-line Transformation

The *B*. *mori* polyvoltine strain with diapause ability, *Lan10*, was used for germ-line transformation according to a previously described method^50,51^ with modifications. To generate transgenic silkworms that expressed continuous repetitive sequences of several desired sizes, high-efficiency transposition of a *piggyBac* transposon by TAL effectors was used in the transgenic experiment. The mRNA of the pESNT-*PBase* vector was also used as the helper plasmid according to a previously described method^52^. The *piggyBac*-derived vector described above was mixed with the mRNA of the pESNT-*PBase* vector or the helper vector at a 1:1 proportion and microinjected into preblastoderm generation 0 (G0) embryos, which were then incubated at 25 °C in a humidified chamber for approximately 10 days until the larvae hatched. Larvae were reared with fresh mulberry leaves under standard conditions (25 °C, 80% R.H).

### Preparation of heterozygous and homozygous transgenic lineages

The positive transgenic individuals were screened at the G1 egg stage through the marker gene (3 × P3-DsRed) expression using fluorescence microscopy (Olympus SZX16, Japan). Both G0 and G1 individuals were mated with WT moths to make each transgenic lineage carry one single insertion site. Each positive transgenic brood was reared together and sib-mated in G2, and the character segregation of Ds-Red expression with a 1:2:1 genotype ratio occurred in G3. The character segregation also existed in the G4 transgenic brood through G3 sequential positive individual sib-mating, and the transgenic brood that had negative individuals would be discarded in G4. Sequential sib-mating of the positive brood was performed until G5, and more than eight individuals for each transgenic brood were chosen for a testcross experiment to verify whether this transgenic lineage was already homozygous. If the resulting G6 individuals were all positive, we could confirm that their parents, from G5 transgenic lineages, were homozygous, i.e., the cocoon silk included the re-MaSp1or re-MaSp2 expressed by two gene copies. The silk fibre of the G5 homozygous transgenic lineages were used for mechanical property testing. Meanwhile, to measure the mechanical properties of silk fibres that contained the re-MaSp1or re-MaSp2 expressed by only one copy gene, positive transgenic individuals were also mated with WT moths in G3, and the resulting G4 positive silkworms were heterozygous. Finally, both the heterozygous and homozygous lineages of each transgenic strain were generated in G4 and G5 through this process, respectively.

### Inverse PCR analysis

The inverse PCR analysis was performed as previously described with minor modifications^50^. Genomic DNA was isolated from the PSGs of DsRed-positive fifth instar larvae in heterozygous G4 according to standard SDS lysis-phenol treatment after incubation with proteinase K, and then was further treated by RNase and purified. DNA was digested with *Sau3A*І at 37 °C for 2 h and then circularized by T_4_ DNA ligase (TaKaRa, China) overnight at 16 °C. PCR amplification was carried out using the circularized fragments as templates under standard conditions with primers designed from the right-arm or the left-arm of the *piggy*Bac vector, including R-inverse 1-F and R-inverse 1-R for the first PCR and R-inverse 2-F and R-inverse 2-R for the second PCR and L-inverse 1-F and L-inverse 1-R for the first PCR and L-inverse 2-F and L-inverse 2-R for the second PCR (Supplementary Table S4). Amplification conditions were performed as follows: 94 °C for 3 min, 35 cycles of 94 °C for 30 s, 60 °C for 30 s, and 72 °C for 3 min, and a final extension period of 72 °C for 10 min. Amplification with the left primers (L-inverse 1-F and L-inverse 1-R; L-inverse 2-F and L-inverse 2-R) was performed under the same conditions except that annealing was carried out at 55 °C. Amplified fragments were sequenced after cloning into the pMD19-T vector. Searches of the silkworm genome database (http://sgp.dna.affrc.go.jp/KAIKObase/) localized transgenes to distinct chromosomes.

### Quantitative real-time PCR (qRT-PCR) analysis

Quantitative real-time PCR (qRT-PCR) analysis was performed to analyze transcripts in transgenic silkworm strains as previously described^53^. The gene-specific primers were designed using Primer Premier 5.0 (Supplementary Table S4). Total RNA samples were extracted from the PSGs on the 3^rd^ day of the fifth instar larvae using TRIzol Reagent (Invitrogen). The cDNA was prepared using primeScript^®^ RT reagent Kit with gDNA Eraser (TaKaRa, China). The PCR products were amplified and detected in real time with the ABI Step-one Real-Time PCR system (Applied Biosystems, CA) using the SYBR^®^*Premix Ex Taq*^TM^ kit (TaKaRa, China) according to the manufacturer’s protocol. The amplification reaction was performed in a 20.0 µl reaction mixture as follows: denaturation at 95°Cfor 30 s followed by 40 cycles of 95°Cfor 5 s and 60 °C for 30 s. Subsequently, melting curves were constructed. The endogenous *B*. *mori* glyceraldehyde-3-phosphate dehydrogenase (*BmGAPDH*, accession number: BGIBMGA007490-TA) was used as a control. A relative quantitative method (threshold cycle[△△Ct]) was used to present the relative expression levels of the detected genes. All samples were tested with three independent replicates.

### Western Blotting

Preparation of the intact solubilized fibroin from cocoon shells was performed according to a previously described method^54^ with some modifications. Briefly, five cocoons for each silkworm strain were degummed by 8 M urea, and the resulting fibroin was dissolved in 9.3 M aqueous lithium thiocyanate (LiSCN) containing 2% (vol/vol) ß-mercaptoethanol (BME) at room temperature, followed by dialysis against deionized water overnight. Insoluble materials from the samples were centrifuged, and then the supernatants were harvested as protein samples for further assays.

Each sample contained 80 µg total protein mixed 1:1 with electrophoresis buffer and was incubated at 50 °C for 10 min, then loaded onto 5%–12% gradient SDS-PAGE gels. After separation, proteins were blotted onto PVDF membrane (Immobilon-P, Millipore) using a GE transfer cell according to the manufacturer’s instructions. The membrane was blocked with 3% BSA in TBS-T (10 mM Tris, 150 Mm NaCl, and 0.1% Tween20), and then incubated withre-MaSp1- or re-MaSp2-specific polyclonal peptide antiserum (1:1000 dilution) as the primary antibody and Peroxidase conjugate goat anti-rabbit IgG-HRP (1:5000 dilution, Jackson) as the secondary antibody. The primary antibodies for immunoblotting analysis included the anti-re-MaSp1 or anti-re-MaSp2 peptide-specific polyclonal rabbit anti-serum antibodies (GenScript). The anti-re-MaSp-specific peptide antibodies were raised in rabbits against the peptides “CSSNIGSVNYDSSGQ” or “CGGAGPGRQQAYGPG”, which corresponds to the re-MaSp1 or re-MaSp2 protein, respectively. Signal detection was performed with the ECL Plus Western Blotting Detection Kit (GE Healthcare).

To further identify recombinant dragline silk protein content in transgenic silk fibres, a relative amount was quantified to estimate the proportions of the targeted protein against the Fib-L protein as previously described^55^. All samples were performed with three independent replicates.

### Mechanical testing of transgenic silk fibres

Five weight-matched cocoons from each silkworm strain were randomly chosen and three pieces of silk fibre at the beginning of each cocoon were used to measure the mechanical properties. Single silk fibres were prepared from the obtained cocoons according to a previously described method^56^. Briefly, to obtain a single silk fibre from a cocoon for tensile tests, each cocoon was first cooked in deionized water for 2 min at 100 °C to vent the air inside the cocoon and was then soaked in 65 °C water for 3 min to saturate all of the cocoon layers. Subsequently, the cocoon was cooked in deionized water for 2 min at 100 °C to soften the sericin and then cooled gradually down to 85 °C for approximately 15 min. The sericin layer swelled during this process. Finally, the cocoon was moved to warm water at65 °C to reel the silk fibre. During this process, precaution was taken to avoid stretching the fibres plastically.

The specimens were prepared as previously described^57^ for tensile tests. Each specimen was carefully glued (glue502) across a rectangular frame, which was cut into a cardboard support to obtain a gauge length of 30 mm. The initial length (*L0*) of each silk fibre was 30 mm. The cross-sectional diameter of each silk sample was measured using a digital microscope (VHX-600, Keyence) at 1000× magnification. To ensure effective averaging and to minimize the possible problem that could affect the measurement of the diameter measured, four measurements were obtained from each sample, and the average diameter was calculated. In this experiment, we calculated the cross-sectional area using this diameter, under the assumption of a circular cross-sectional shape for the cocoon silk fibre. Because the measured diameters may include the thin sericin layer, the diameters and the cross-sectional areas were consistently overestimated compared with the real silk fibre. Thus, the values for the mechanical properties might have been underestimated relative to the real silk fibres.

The tests were performed under ambient conditions (25 ± 2 °C and 60 ± 5% humidity) using an AGS-J Universal Test instrument (Shimadzu Ltd, Japan) equipped with a 5-N load cell at a constant speed of 2 mm/min and frequency of 250 MHz. In the experiments, the specimens were equilibrated under the described conditions for 24 h prior to tensile tests. Cardboard supports with the attached silk were loaded into the AGS-J Universal Test instrument. Both sides of the cardboard frame were carefully cut with ascissor prior to initiating a test, such a way that the force was subsequently transmitted through the silk fibre. The load-displacement data sets were recorded automatically with the control software (Trapezium2, Shimadzu). The values of the toughness were calculated from the area under the stress-strain curves, by integrating the area using mathematical software (Excel, Microsoft; Origin85). In this study, 15 specimens were used for the wild-type silkworm strain. The sample number for the MASP1-2 transgenic silkworm lineages was 30, and that for the other three, including MASP1-12, MASP1-16 and MASP2-24, was 60 samples each.

### Field emission scanning electron microscopy of the composite silk fibres

The fracture surfaces were obtained after brittle fracturing in liquid nitrogen, and the fibres were used for surface observation. The surface and cross sections of the fibres were placed on scanning electron microscopy stubs, coated with platinum at an accelerating voltage of 2 kV for two minutes, observed and photographed using a field emission scanning electron microscope (SU8010, Hitachi, Japan).

### Statistical analyses

Statistical analysis was performed using two-tailed Student’s t-test to determine if the averages were significantly different among the transgenic silkworm lineages. The relationship between the protein length and mechanical properties was assessed through correlation analysis and liner regression analysis using Excel or SPSS software.

## Electronic supplementary material

Supplementary Information

## Data Availability

All data generated of analyzed during this study are included in this published article, and its Supplementary Information files.
