# Supplementary material for: Extraordinary Mechanical Properties of Composite Silk Through Hereditable Transgenic Silkworm Expressing Recombinant Major Ampullate Spidroin
Source: Sci Rep. 2018 Oct 29;8:15956. doi: 10.1038/s41598-018-34150-y (PMC6206087; doi:10.1038/s41598-018-34150-y)
Supplement: Supplementary file 1 — Supplementary Information [file 41598_2018_34150_MOESM1_ESM.pdf]

Extraordinary Mechanical Properties of Composite Silk Through Hereditable Transgenic Silkworm Expressing Recombinant Major Ampullate Spidroin

Zhengying You[1], Xiaogang Ye[1], Lupeng Ye[1], Qiujie Qian[1], Meiyu Wu[1], Jia Song[1], Jiaqian Che[1] and Boxiong Zhong[1,*]

1 College of Animal Science, Zhejiang University, Hangzhou 310058, P. R. China

* Corresponding author: Email: bxzhong@zju.edu.cn.

This file includes:

Supporting Information

Supplementary Figures S1-S7

Supplementary Table S1-S4

Supplementary Data S1-S2

The full-length SDS-PAGE gels and blots of Figure 4 and Supplementary Figure S6

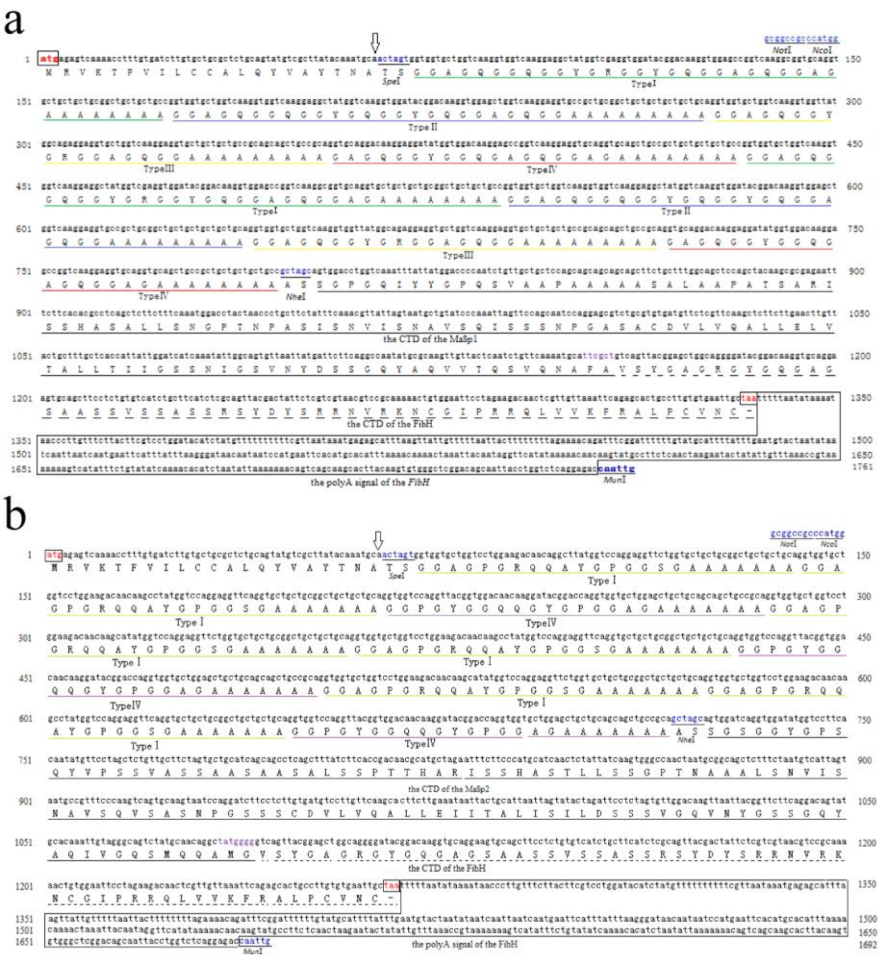

c

Vectors	Length of re-MaSp1/2 sequences（bp）	The type and number of repetitive units	Length of repetitive sequences（bp）	Predicated MW (kDa)
pBac[3xP3-DsRed]-MaSp1x2	1257	Type(1-2-3-4) / 2	726	39.2
pBac[3xP3-DsRed]-MaSp1x12	4923	Type (1-2-3-4) / 12	4392	133.5
pBac[3xP3-DsRed]-MaSp1x16	6387	Type (1-2-3-4) / 16	5856	171.2
pBac[3xP3-DsRed]-MaSp2x24	5772	Type (1'-1'-4') / 24	5232	161.6

Supplementary Figure S1 The designed segments of re-MaSp1 and re-MaSp2

(a) The sequence of designed segments, including two typical repetitive units of MaSp1, was sub-cloned into the pUC57-MaSp1 vector. **(b)**The sequence of designed segments, including three typical repetitive units of MaSp2, was sub-cloned into the Puc57-MaSp2 vector. (**c**)The information for the four transgenic vectors used in this study.

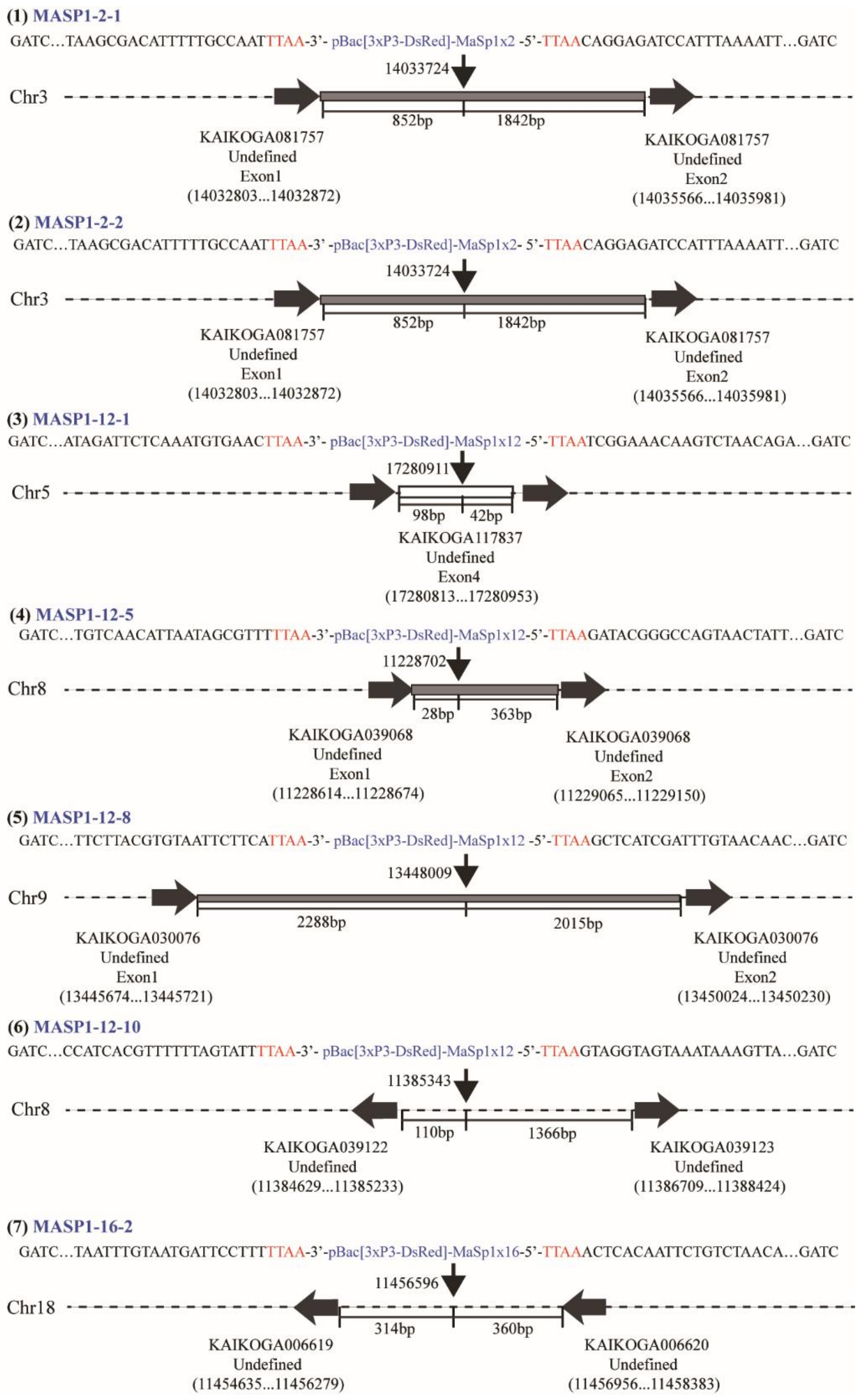
(1) MASP1-2-1
GATC…TAAGCGACATTTTTGCCAATTTAA-3'- pBac[3xP3-DsRed]-MaSp1x2 -5'-TTAACAGGAGATCCATTTAAAATT…GATC
14033724
Chr3
852bp
1842bp
KAIKOGA081757
Undefined
Exon1
(14032803...14032872)
KAIKOGA081757
Undefined
Exon2
(14035566...14035981)
(2) MASP1-2-2
GATC…TAAGCGACATTTTTGCCAATTTAA-3' -pBac[3xP3-DsRed]-MaSp1x2- 5'-TTAACAGGAGATCCATTTAAAATT…GATC
14033724
Chr3
852bp
1842bp
KAIKOGA081757
Undefined
Exon1
(14032803...14032872)
KAIKOGA081757
Undefined
Exon2
(14035566...14035981)
(3) MASP1-12-1
GATC…ATAGATTCTCAAATGTGAACTTAA-3'- pBac[3xP3-DsRed]-MaSp1x12 -5'-TTAATCGGAAACAAGTCTAACAGA…GATC
17280911
Chr5
98bp
42bp
KAIKOGA117837
Undefined
Exon4
(17280813...17280953)
(4) MASP1-12-5
GATC…TGTCAACATTAATAGCGTTTTTAA-3'-pBac[3xP3-DsRed]-MaSp1x12-5'-TTAAGATACGGGCCAGTAACTATT…GATC
11228702
Chr8
28bp
363bp
KAIKOGA039068
Undefined
Exon1
(11228614...11228674)
KAIKOGA039068
Undefined
Exon2
(11229065...11229150)
(5) MASP1-12-8
GATC…TTCTTACGTGTAATTCTTCATTAA-3'- pBac[3xP3-DsRed]-MaSp1x12 -5'-TTAAGCTCATCGATTTGTAACAAC…GATC
13448009
Chr9
2288bp
2015bp
KAIKOGA030076
Undefined
Exon1
(13445674...13445721)
KAIKOGA030076
Undefined
Exon2
(13450024...13450230)
(6) MASP1-12-10
GATC…CCATCACGTTTTTTAGTATTTTAA-3'- pBac[3xP3-DsRed]-MaSp1x12 -5'-TTAAGTAGGTAGTAAATAAAGTTA…GATC
11385343
Chr8
110bp
1366bp
KAIKOGA039122
Undefined
(11384629...11385233)
KAIKOGA039123
Undefined
(11386709...11388424)
(7) MASP1-16-2
GATC…TAATTTGTAATGATTCCTTTTTAA-3'-pBac[3xP3-DsRed]-MaSp1x16-5'-TTAAACTCACAATTCTGTCTAACA…GATC
11456596
Chr18
314bp
360bp
KAIKOGA006619
Undefined
(11454635...11456279)
KAIKOGA006620
Undefined
(11456956...11458383)

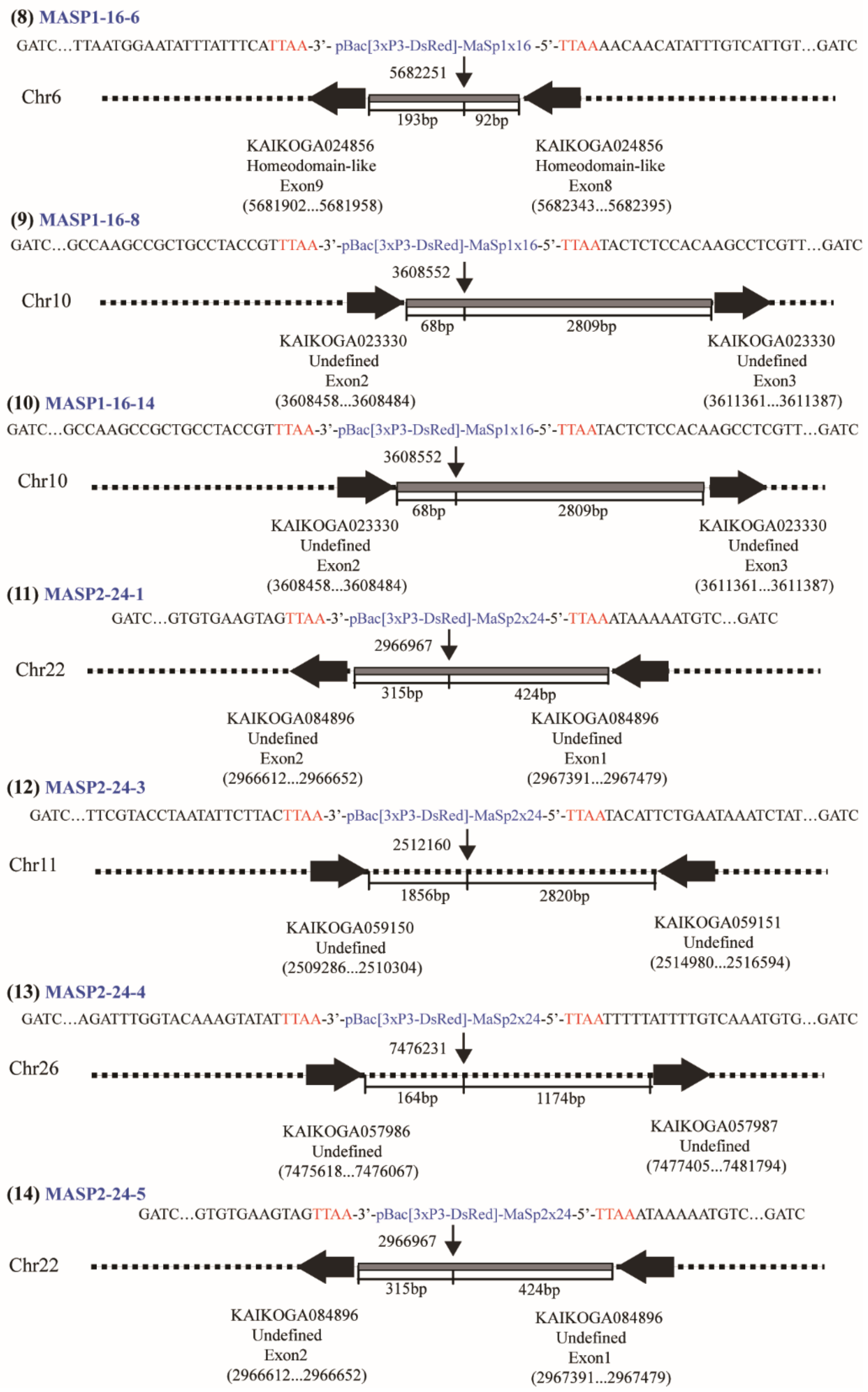

Supplementary Figure S2 Genomic insertion sites of the 14 transgenic lineages

MASP1-2-1 and MASP1-2-2 were the transgenic silkworm lineages harboring the 2-fold typical repetitive units of MaSp1; MASP1-12-1, MASP1-12-5, MASP1-12-8 and MASP1-12-10 were the transgenic silkworm lineages harboring the 12- fold typical repetitive units of MaSp1 with different insertion sites; MASP1-16-2 was the transgenic silkworm lineages harboring the 16- fold typical repetitive units of MaSp1

with different insertion sites.MASP1-16-6, MASP1-16-8 and MASP1-16-14 were the transgenic silkworm lineages harboring the 16- fold typical repetitive units of MaSp1 but with different insertion sites; MASP2-24-1, MASP2-24-3, MASP2-24-4 and MASP2-24-5 were the transgenic silkworm lineages harboring the 24- fold typical repetitive units of MaSp2 but with different insertion sites.

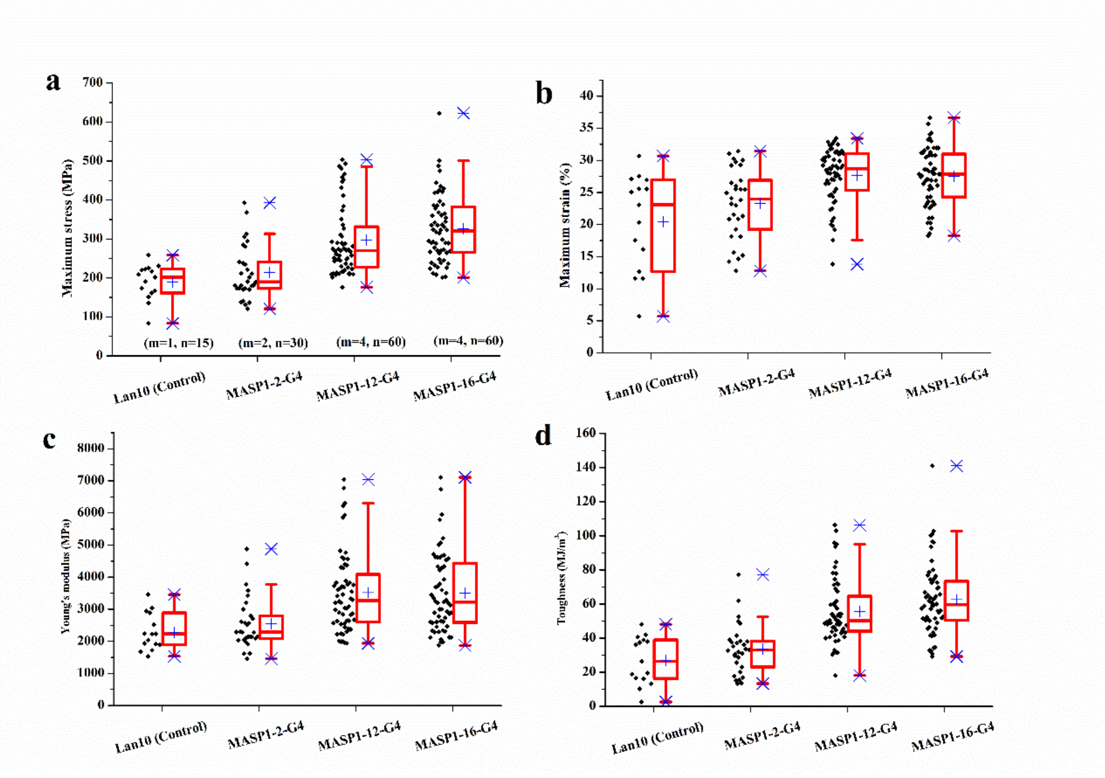

Supplementary Figure S3 Comparison analysis of the mechanical properties of the composite silk fibres in heterozygous G4

The mechanical properties are shown for the composite silk fibres, including Maximum stress (a), Maximum strain (b), Young's modulus(c) and Toughness(d) for *Lan10* (Control), MASP1-2-G4, MASP1-12-G4 and MASP1-16-G4. The average value, median, 25-75% group and the entire range are shown; detailed data are given in Tables S3. The number of silk fibres is shown in the Maximum stress graph. n: number of transgenic silkworm lineage, m: number of silk fibres.

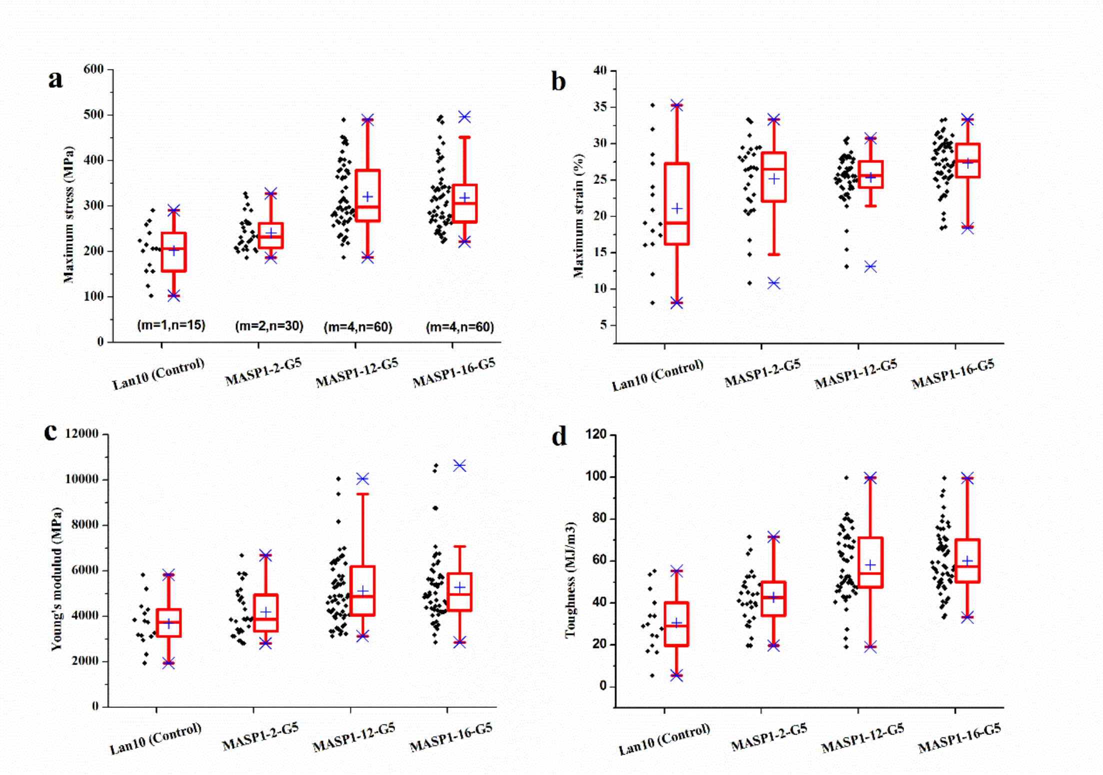

Supplementary Figure S4 Comparison analysis of the mechanical properties of the composite silk fibres in homozygous G5

The mechanical properties are shown for the composite silk fibres, including Maximum stress **(a)**, Maximum strain **(b)**, Young's modulus **(c)** and Toughness **(d)** for *Lan10* (Control), MASP1-2-G5, MASP1-12-G5 and MASP1-16-G5. The average value, median, 25-75% group and the entire range are shown; detailed data are given in Tables S4. The number of silk fibres is shown in the Maximum stress graph. n: number of transgenic silkworm lineage, m: number of silk fibres.

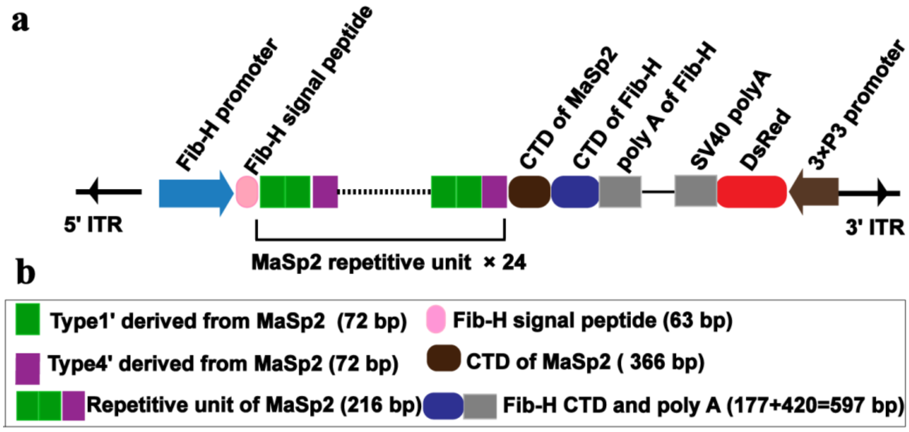

Supplementary Figure S5 Schematic representation for expression cassettes of the pBac[3×P3-DsRed]-MaSp1×24 transgenic vectors

(**a**) Schematic of the pBac[3×P3-DsRed]-MaSp1×24 vector was used to express re-MaSp2 including 24-fold repetitive units of MaSp2. (**b**) The key elements are shown using different color boxes. Fib-H promoter: the primary promoter of *Fib-H*(1269bp); SP: the signal peptideof *Fib-H*; CTD of MaSp2: C-terminal domain of the *MaSp2*; Fib-HCTD and polyA: the partial C-terminal domain and polyA signal of the *Fib-H*; and 3×P3-DsRed was used as the marker genefor screening positive individuals.

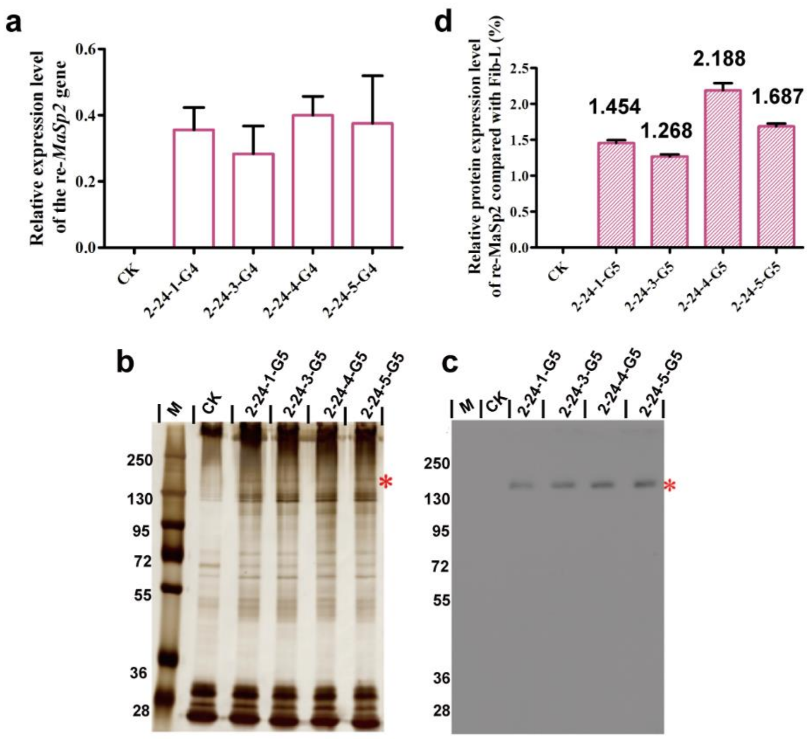

Supplementary Figure S6 The expression analysis of exogenous re-MaSp2

(**a**) The relative expression analysis of *re-MaSp2* in the PSGs of the 3rd day of the fifth instar in G4 heterozygous transgenic lineages was performed by qRT-PCR. Mean ± SD were derived from three experiments. (**b**)The gradient sodium dodecyl sulfate-polyacrylamide gel (5%-12%SDS-PAGE) analysis of the degummed silkworm cocoons was performed and (**c**) followed by immunoblotting on nitrocellulose membranes; M: 250 kDa protein Marker; CK: the degummed silkworm cocoons of wide-type *Lan10*, the degummed silkworm cocoons of MASP2-24-1-G5, MASP2-24-3-G5, MASP2-24-4-G5 and MASP2-24-5-G5 transgenic lineages. (**d**) Expression analysis of the re-MaSp2 expression relative to the Fib-L protein in homozygous G5 through gray analysis using Gel-Pro-analyzer4 software. Mean ± SD were derived from three experiments.

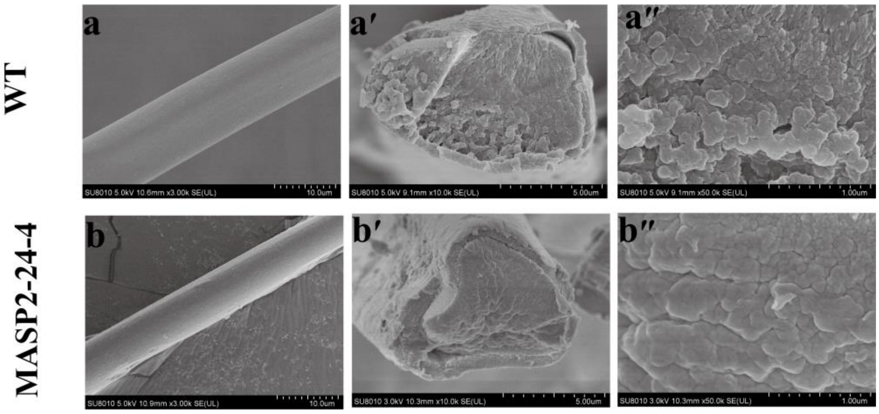

Supplementary Figure S7 Field emission scanning electron micrographs of the fibres

The surface structure of the silk fibres derived from the non-transgenic silkworm lineages **(a)** and the transgenic silkworm lineages MASP2-24-2**(b)**; The cross section structure of the silk fibres derived from the non-transgenic silkworm lineages **(a' and a'')** and the transgenic silkworm lineages MASP2-24-2 **(b' and b'')**. Scale bars in a-b, 10μm; in a'-b', 5μm; in a''-b'', 1μm.

Supplementary Table S1 Microinjection of the four *piggy*Bac-derived vectors in silkworm embryos of the Lan10 strain

Vectors	Transgenic lineages	Injected embryos (G0)	Hatched embryos (G0)	Hatched (%)	G1 broods	Positive brood	G1 positive (%)
**Microinjection with helper vector**							
**pBac[3×P3-DsRed]-MaSp1×2 / Helper**	**MASP1-2**	1100	260	23.64	45	2	4.44
**pBac[3×P3-DsRed]-MaSp1×12 / Helper**	**MASP1-12**	1200	186	15.5	70	1	1.43
**pBac[3×P3-DsRed]-MaSp1×16 / Helper**	**MASP1-16**	2850	676	23.72	138	1	0.72
**pBac[3×P3-DsRed]-MaSp2×24 / Helper**	**MASP2-24**	3100	859	27.71	90	1	1.11
**Microinjection with mRNA of pESNT-Pbase vector**							
**pBac[3×P3-DsRed]-MaSp1×12/pESNT-Pbase**	**MASP1-12**	1100	370	33.64	106	10	9.43
**pBac[3×P3-DsRed]-MaSp1×16/pESNT-Pbase**	**MASP1-16**	1000	330	33	109	16	14.68
**pBac[3×P3-DsRed]-MaSp2×24 /pESNT-Pbase**	**MASP2-24**	1000	279	27.9	51	5	9.8

Supplementary Table S2 Mechanical properties of the composite silk fibres from the transgenic silkworm in heterozygous G4

Transgenic lineages	Maximum stress (MPa)	SD	Improved fold	Maximum strain (%)	SD	Improved fold	Young's modulus (MPa)	SD	Improved fold	Toughness (MJ/m3)	SD	Improved fold
**Lan10 (Control)**	189.891	44.865		20.482	7.481		2283.28	568.1		27.01	13.947	
**MASP1-2-1-G4**	211.923	72.956		23.605	5.713		2700.76	1056		34.208	17.43	
**MASP1-2-2-G4**	216.704	70.329		22.963	5.567		2406.03	487.8		32.883	12.489	
**Average**	**214.314**		**1.129**	**23.284**		**1.137**	**2553.395**		**1.118**	**33.545**		**1.242**
**MASP1-12-1-G4**	274.621	67.064		25.989	5.118		3670.91	1275		49.25	15.709	
**MASP1-12-5-G4**	305.352	104.76		28.084	3.604		3469.47	1156		57.285	18.54	
**MASP1-12-8-G4**	311.416	96.322		29.037	3.36		3714.33	1393		62.186	20.113	
**MASP1-12-10-G4**	295.967	96.373		26.866	4.232		3204.14	1175		53.659	19.942	
**Average**	**296.839**		**1.563**	**27.471**		**1.341**	**3514.713**		**1.539**	**55.595**		**2.058**
**MASP1-16-2-G4**	336.263	86.091		29.45	2.497		3817.28	1481		68.724	20.115	
**MASP1-16-6-G4**	345.039	100.02		27.515	3.968		3770.04	1260		68.004	26.426	
**MASP1-16-8-G4**	298.395	77.198		25.286	4.61		3518.07	917.5		52.319	15.242	
**MASP1-16-14-G4**	326.199	78.832		27.951	5.208		3308.45	1200		61.253	17.529	
**Average**	**326.474**		**1.719**	**27.55**		**1.345**	**3603.46**		**1.578**	**62.575**		**2.317**
**MASP2-24-1-G4**	302.916	85.075		27.344	3.421		3129.4	1331		55.222	18.533	
**MASP2-24-3-G4**	320.949	67.405		25.104	4.541		2781.23	1075		53.348	16.097	
**MASP2-24-4-G4**	307.941	58.525		27.116	3.708		3193.5	1047		56.16	13.407	
**MASP2-24-5-G4**	278.147	54.893		24.737	3.757		3915.86	1516		46.702	10.904	
**Average**	**302.488**		**1.593**	**26.075**		**1.273**	**3255.005**		**1.426**	**52.858**		**1.957**

Supplementary Table S3 Mechanical properties of the composite silk fibres from the transgenic silkworm in homozygous G5

Transgenic lineages	Maximum stress (MPa)	SD	Improved fold	Maximum strain (%)	SD	Improved fold	Young's modulus (MPa)	SD	Improved fold	Toughness (MJ/m3)	SD	Improved fold
**Lan10 (Control)**	202.027	52.47		21.149	7.357		3681.16	1019		30.573	13.972	
**MASP1-2-1-G5**	234.135	31.903		23.169	5.742		4452.62	1235		41.466	11.214	
**MASP1-2-2-G5**	247.95	42.065		23.237	4.794		3904.22	833.6		40.968	17.977	
**Average**	**241.043**		**1.193**	**23.203**		**1.097**	**4178.42**		**1.135**	**41.217**		**1.348**
**MASP1-12-1-G5**	328.76	74.123		25.086	4.075		4912.51	1576		59.033	18.593	
**MASP1-12-5-G5**	332.727	81.344		25.314	2.89		5186.58	1050		59.494	15.495	
**MASP1-12-8-G5**	320.916	48.726		26.152	2.008		5237.27	1334		59.315	9.297	
**MASP1-12-10-G5**	301	82.529		24.905	3.332		5120.63	1915		54.443	19.283	
**Average**	**320.851**		**1.588**	**25.364**		**1.199**	**5114.248**		**1.389**	**58.071**		**1.899**
**MASP1-16-2-G5**	343.044	73.626		28.037	2.575		5457.54	1371		66.634	14.567	
**MASP1-16-6-G5**	349.619	85.833		27.902	2.603		5431.69	2201		66.463	14.155	
**MASP1-16-8-G5**	303.534	43.534		25.271	4.561		4597.31	1042		48.826	12.038	
**MASP1-16-14-G5**	307.611	44.76		28.063	3.158		5617.37	1252		58.871	11.026	
**Average**	**325.951**		**1.613**	**27.318**		**1.292**	**5275.978**		**1.433**	**60.199**		**1.969**
**MASP2-24-1-G5**	332.351	71.094		27.788	5.709		4747.72	1363		63.694	19.145	
**MASP2-24-3-G5**	314.108	92.934		25.626	3.511		5495.9	1132		56.981	21.248	
**MASP2-24-4-G5**	303.674	88.296		25.363	3.906		5481.1	1941		53.817	19.459	
**MASP2-24-5-G5**	305.387	46.187		27.872	5.354		5014.95	1154		59.404	15.83	
**Average**	**313.88**		**1.554**	**26.662**		**1.261**	**5184.918**		**1.409**	**58.474**		**1.913**

Supplementary Table S4 The primers used in this study

Primer name	Sequence (5'-3')	Length of the products (bp)	Purpose
**pFib-H-F**	CTCGAGCAGCTCTAACACTAGGCTAAC	1269	PCR for the primary promoter of the Fib-H gene (*Xho*I and *Nco*I site are underlined)
**pFib-H-R**	CCATGGCTTGAGAGTTGGAACCG		
**R-inverse 1-F**	TCTGTATATCGAGGTTTATTTA	depended on the inverse site	the first primers for the inverse PCR of the right arm of the *piggy*Bac vector
**R-inverse 1-R**	CCGATAAAAACACATGC		
**R-inverse 2-F**	ACTCAAAATTTCTTCTAAAGTAACAA	depended on the inverse site	the second primers for the inverse PCR of the right arm of the *piggy*Bac vector
**R-inverse 2-R**	CTTTAACGTACGTCACAATATG		
**L-inverse 1-F**	GACAAGCACGCCTCAGCC	depended on the inverse site	the first primers for the inverse PCR of the left arm of the *piggy*Bac vector
**L-inverse 1-R**	TGAGTCAAAATGACGCATGATTATC		
**L-inverse 2-F**	GCTCCAAGCGGCGACTG	depended on the inverse site	the second primers for the inverse PCR of the left arm of the *piggy*Bac vector
**L-inverse 2-R**	GGGATGTTCTTTAGACGATGAGC		
**MaSp1-F**	TTCTTTCAAATGGACCTACTAACC	1357	PCR for specific identification of the *re-MaSp1*fregment
**MaSp1-R**	CCGGCTACTACTACGTGGACTC		
**MaSp2-F**	CTTTATCTTCACCGACAACGC	1358	PCR for specific identification of the *re-MaSp2*fregment
**MaSp2-R**	GACTACACCATCGTGGAGCAG		
**GAPDH-qF**	GAAAAGGGAGCTCAAGTGGTCGC	133	qPCR for *GAPDH* gene
**GAPDH-qR**	CAACAAGGAATCCATCCTGAACCTC		
**MaSp1-q F**	TTCAAATGGACCTACTAACCCTGC	84	qPCR for *re-MaSp*1 gene
**MaSp1-q R**	GCTCCTGGATTGCTGGAACTAA		
**MaSp2-q F**	GCTTTATCTTCACCGACAACGC	97	qPCR for *re-MaSp2* gene
**MaSp2-q R**	TGACATTAGAAAGAGCTGCCGC		

Supplementary information data

Supplementary data S1 The key component sequence of pBac[3×P3-DsRed]-MaSp1×16 expression vector.

>The key component sequence of pBac[3×P3-DsRed]-MaSp1×16 expression vector

Fib-H promoter

Fib-H signal peptide

MaSp1 repetitive unit×16

CTD of MaSp1

CTD of Fib-H and Poly A of Fib-H

The partial SV40

SV40 poly A-DsRed-3×P3 promoter

The key component sequences were marked with the corresponding colour.

CTCGAGCAGCTCTAACACTAGGCTAACTCAGGCTTAGTAGCCTGGTCCTAGTGTTAGATT
TGAAGTCGTCTAATGCAAAGATTATTGGATCTGATGGATCCGTAAGGACGTGTCTAGAGC
GTCGACGGTGACTAGCTCCTGCGTGATCAGGAAAAATGTGGAAAGCTTAACGATTTTGTC
ACATTTTACTTATCACAACTTGTTTTTATAATAATTCGCTTAAATGAGCAGCTATTACTT
AATCTCGTAGTGGTTTTTGACAAAATCAGCTTCTTTAGAACTAAAATATCATTTTTTTCG
TAATTTTTTTAATGAAAAATGCTCTAGTGTTATACCTTTCCAAAATCACCATTAATTAGG
TAGTGTTTAAGCTTGTTGTACAAAACTGCCACACGCATTTTTTTCTCCACTGTAGGTTGT
AGTTACGCGAAAACAAAATCGTTCTGTGAAAATTCAAACAAAAATATTTTTTCGTAAAAA
CACTTATCAATGAGTAAAGTAACAATTCATGAATAATTTCATGTAAAAAAAAAATACTAG
AAAAGGAATTTTTCATTACGAGATGCTTAAAAATCTGTTTCAAGGTAGAGATTTTTCGAT
ATTTCGGAAAATTTTGTAAAACTGTAAATCCGTAAAATTTTGCTAAACATATATTGTGTT
GTTTTGGTAAGTATTGACCCAAGCTATCACCTCCTGCAGTATGTCGTGCTAATTACTGGA
CACATTGTATAACAGTTCCACTGTATTGACAATAATAAAACCTCTTCATTGACTTGAGAA
TGTCTGGACAGATTTGGCTTTGTATTTTTGATTTACAAATGTTTTTTTGGTGATTTACCC
ATCCAAGGCATTCTCCAGGATGGTTGTGGCATCACGCCGATTGGCAAACAAAAACTAAAA
TGAAACTAAAAAGAAACAGTTTCCGCTGTCCCGTTCCTCTAGTGGGAGAAAGCATGAAGT
AAGTTCTTTAAATATTACAAAAAAATTGAACGATATTATAAAATTCTTTAAAATATTAAA
AGTAAGAACAATAAGATCAATTAAATCATAATTAATCACATTGTTCATGATCACAATTTA
ATTTACTTCATACGTTGTATTGTTATGTTAAATAAAAAGATTAATTTCTATGTAATTGTA
TCTGTACAATACAATGTGTAGATGTTTATTCTATCGAAAGTAAATACGTCAAAACTCGAA
AATTTTCAGTATAAAAAGGTTCAACTTTTTCAAATCAGCATCAGTTCGGTTCCAACTCTC
AAGCCATGGATGAGAGTCAAAACCTTTGTGATCTTGTGCTGCGCTCTGCAGTATGTCGCT
TATACAAATGCAACTAGTGGTGGTGCTGGTCAAGGTGGTCAAGGAGGCTATGGTCGAGG
TGGATACGGACAAGGTGGAGCCGGTCAAGGCGGTGCAGGTGCTGCTGCTGCGGCTGCTGC
TGCCGGTGGTGCTGGTCAAGGTGGTCAAGGAGGCTATGGTCAAGGTGGATACGGACAAGG
TGGAGCTGGTCAAGGAGGTGCCGCTGCGGCTGCTGCTGCTGCTGCAGGTGGTGCTGGTCA
AGGTGGTTATGGCAGAGGAGGTGCTGGTCAAGGAGGTGCTGCTGCTGCCGCAGCAGCTGC
CGCAGGTGCAGGACAAGGAGGATATGGTGGACAAGGAGCCGGTCAAGGAGGTGCAGGTGC

AGCTGCCGCTGCTGCTGCTGCCGGTGGTGCTGGTCAAGGTGGTCAAGGAGGCTATGGTCG
AGGTGGATACGGACAAGGTGGAGCCGGTCAAGGCGGTGCAGGTGCTGCTGCTGCGGCTGC
TGCTGCCGGTGGTGCTGGTCAAGGTGGTCAAGGAGGCTATGGTCAAGGTGGATACGGACA
AGGTGGAGCTGGTCAAGGAGGTGCCGCTGCGGCTGCTGCTGCTGCTGCAGGTGGTGCTGG
TCAAGGTGGTTATGGCAGAGGAGGTGCTGGTCAAGGAGGTGCTGCTGCTGCCGCAGCAGC
TGCCGCAGGTGCAGGACAAGGAGGATATGGTGGACAAGGAGCCGGTCAAGGAGGTGCAGG
TGCAGCTGCCGCTGCTGCTGCTGCCGCTAGTGGTGGTGCTGGTCAAGGTGGTCAAGGAGG
CTATGGTCGAGGTGGATACGGACAAGGTGGAGCCGGTCAAGGCGGTGCAGGTGCTGCTGC
TGCGGCTGCTGCTGCCGGTGGTGCTGGTCAAGGTGGTCAAGGAGGCTATGGTCAAGGTGG
ATACGGACAAGGTGGAGCTGGTCAAGGAGGTGCCGCTGCGGCTGCTGCTGCTGCTGCAGG
TGGTGCTGGTCAAGGTGGTTATGGCAGAGGAGGTGCTGGTCAAGGAGGTGCTGCTGCTGC
CGCAGCAGCTGCCGCAGGTGCAGGACAAGGAGGATATGGTGGACAAGGAGCCGGTCAAGG
AGGTGCAGGTGCAGCTGCCGCTGCTGCTGCTGCCGGTGGTGCTGGTCAAGGTGGTCAAGG
AGGCTATGGTCGAGGTGGATACGGACAAGGTGGAGCCGGTCAAGGCGGTGCAGGTGCTGC
TGCTGCGGCTGCTGCTGCCGGTGGTGCTGGTCAAGGTGGTCAAGGAGGCTATGGTCAAGG
TGGATACGGACAAGGTGGAGCTGGTCAAGGAGGTGCCGCTGCGGCTGCTGCTGCTGCTGC
AGGTGGTGCTGGTCAAGGTGGTTATGGCAGAGGAGGTGCTGGTCAAGGAGGTGCTGCTGC
TGCCGCAGCAGCTGCCGCAGGTGCAGGACAAGGAGGATATGGTGGACAAGGAGCCGGTCA
AGGAGGTGCAGGTGCAGCTGCCGCTGCTGCTGCTGCCGCTAGTGGTGGTGCTGGTCAAGG
TGGTCAAGGAGGCTATGGTCGAGGTGGATACGGACAAGGTGGAGCCGGTCAAGGCGGTGC
AGGTGCTGCTGCTGCGGCTGCTGCTGCCGGTGGTGCTGGTCAAGGTGGTCAAGGAGGCTA
TGGTCAAGGTGGATACGGACAAGGTGGAGCTGGTCAAGGAGGTGCCGCTGCGGCTGCTGC
TGCTGCTGCAGGTGGTGCTGGTCAAGGTGGTTATGGCAGAGGAGGTGCTGGTCAAGGAGG
TGCTGCTGCTGCCGCAGCAGCTGCCGCAGGTGCAGGACAAGGAGGATATGGTGGACAAGG
AGCCGGTCAAGGAGGTGCAGGTGCAGCTGCCGCTGCTGCTGCTGCCGGTGGTGCTGGTCA
AGGTGGTCAAGGAGGCTATGGTCGAGGTGGATACGGACAAGGTGGAGCCGGTCAAGGCGG
TGCAGGTGCTGCTGCTGCGGCTGCTGCTGCCGGTGGTGCTGGTCAAGGTGGTCAAGGAGG
CTATGGTCAAGGTGGATACGGACAAGGTGGAGCTGGTCAAGGAGGTGCCGCTGCGGCTGC
TGCTGCTGCTGCAGGTGGTGCTGGTCAAGGTGGTTATGGCAGAGGAGGTGCTGGTCAAGG
AGGTGCTGCTGCTGCCGCAGCAGCTGCCGCAGGTGCAGGACAAGGAGGATATGGTGGACA
AGGAGCCGGTCAAGGAGGTGCAGGTGCAGCTGCCGCTGCTGCTGCTGCCGCTAGTGGTGG
TGCTGGTCAAGGTGGTCAAGGAGGCTATGGTCGAGGTGGATACGGACAAGGTGGAGCCGG
TCAAGGCGGTGCAGGTGCTGCTGCTGCGGCTGCTGCTGCCGGTGGTGCTGGTCAAGGTGG
TCAAGGAGGCTATGGTCAAGGTGGATACGGACAAGGTGGAGCTGGTCAAGGAGGTGCCGC
TGCGGCTGCTGCTGCTGCTGCAGGTGGTGCTGGTCAAGGTGGTTATGGCAGAGGAGGTGC
TGGTCAAGGAGGTGCTGCTGCTGCCGCAGCAGCTGCCGCAGGTGCAGGACAAGGAGGATA
TGGTGGACAAGGAGCCGGTCAAGGAGGTGCAGGTGCAGCTGCCGCTGCTGCTGCTGCCGG
TGGTGCTGGTCAAGGTGGTCAAGGAGGCTATGGTCGAGGTGGATACGGACAAGGTGGAGC
CGGTCAAGGCGGTGCAGGTGCTGCTGCTGCGGCTGCTGCTGCCGGTGGTGCTGGTCAAGG
TGGTCAAGGAGGCTATGGTCAAGGTGGATACGGACAAGGTGGAGCTGGTCAAGGAGGTGC
CGCTGCGGCTGCTGCTGCTGCTGCAGGTGGTGCTGGTCAAGGTGGTTATGGCAGAGGAGG
TGCTGGTCAAGGAGGTGCTGCTGCTGCCGCAGCAGCTGCCGCAGGTGCAGGACAAGGAGG
ATATGGTGGACAAGGAGCCGGTCAAGGAGGTGCAGGTGCAGCTGCCGCTGCTGCTGCTGC
CGCTAGTGGTGGTGCTGGTCAAGGTGGTCAAGGAGGCTATGGTCGAGGTGGATACGGACA

AGGTGGAGCCGGTCAAGGCGGTGCAGGTGCTGCTGCTGCGGCTGCTGCTGCCGGTGGTGC
TGGTCAAGGTGGTCAAGGAGGCTATGGTCAAGGTGGATACGGACAAGGTGGAGCTGGTCA
AGGAGGTGCCGCTGCGGCTGCTGCTGCTGCTGCAGGTGGTGCTGGTCAAGGTGGTTATGG
CAGAGGAGGTGCTGGTCAAGGAGGTGCTGCTGCTGCCGCAGCAGCTGCCGCAGGTGCAGG
ACAAGGAGGATATGGTGGACAAGGAGCCGGTCAAGGAGGTGCAGGTGCAGCTGCCGCTGC
TGCTGCTGCCGGTGGTGCTGGTCAAGGTGGTCAAGGAGGCTATGGTCGAGGTGGATACGG
ACAAGGTGGAGCCGGTCAAGGCGGTGCAGGTGCTGCTGCTGCGGCTGCTGCTGCCGGTGG
TGCTGGTCAAGGTGGTCAAGGAGGCTATGGTCAAGGTGGATACGGACAAGGTGGAGCTGG
TCAAGGAGGTGCCGCTGCGGCTGCTGCTGCTGCTGCAGGTGGTGCTGGTCAAGGTGGTTA
TGGCAGAGGAGGTGCTGGTCAAGGAGGTGCTGCTGCTGCCGCAGCAGCTGCCGCAGGTGC
AGGACAAGGAGGATATGGTGGACAAGGAGCCGGTCAAGGAGGTGCAGGTGCAGCTGCCGC
TGCTGCTGCTGCCGCTAGTGGTGGTGCTGGTCAAGGTGGTCAAGGAGGCTATGGTCGAGG
TGGATACGGACAAGGTGGAGCCGGTCAAGGCGGTGCAGGTGCTGCTGCTGCGGCTGCTGC
TGCCGGTGGTGCTGGTCAAGGTGGTCAAGGAGGCTATGGTCAAGGTGGATACGGACAAGG
TGGAGCTGGTCAAGGAGGTGCCGCTGCGGCTGCTGCTGCTGCTGCAGGTGGTGCTGGTCA
AGGTGGTTATGGCAGAGGAGGTGCTGGTCAAGGAGGTGCTGCTGCTGCCGCAGCAGCTGC
CGCAGGTGCAGGACAAGGAGGATATGGTGGACAAGGAGCCGGTCAAGGAGGTGCAGGTGC
AGCTGCCGCTGCTGCTGCTGCCGGTGGTGCTGGTCAAGGTGGTCAAGGAGGCTATGGTCG
AGGTGGATACGGACAAGGTGGAGCCGGTCAAGGCGGTGCAGGTGCTGCTGCTGCGGCTGC
TGCTGCCGGTGGTGCTGGTCAAGGTGGTCAAGGAGGCTATGGTCAAGGTGGATACGGACA
AGGTGGAGCTGGTCAAGGAGGTGCCGCTGCGGCTGCTGCTGCTGCTGCAGGTGGTGCTGG
TCAAGGTGGTTATGGCAGAGGAGGTGCTGGTCAAGGAGGTGCTGCTGCTGCCGCAGCAGC
TGCCGCAGGTGCAGGACAAGGAGGATATGGTGGACAAGGAGCCGGTCAAGGAGGTGCAGG
TGCAGCTGCCGCTGCTGCTGCTGCCGCTAGTGGTGGTGCTGGTCAAGGTGGTCAAGGAGG
CTATGGTCGAGGTGGATACGGACAAGGTGGAGCCGGTCAAGGCGGTGCAGGTGCTGCTGC
TGCGGCTGCTGCTGCCGGTGGTGCTGGTCAAGGTGGTCAAGGAGGCTATGGTCAAGGTGG
ATACGGACAAGGTGGAGCTGGTCAAGGAGGTGCCGCTGCGGCTGCTGCTGCTGCTGCAGG
TGGTGCTGGTCAAGGTGGTTATGGCAGAGGAGGTGCTGGTCAAGGAGGTGCTGCTGCTGC
CGCAGCAGCTGCCGCAGGTGCAGGACAAGGAGGATATGGTGGACAAGGAGCCGGTCAAGG
AGGTGCAGGTGCAGCTGCCGCTGCTGCTGCTGCCGGTGGTGCTGGTCAAGGTGGTCAAGG
AGGCTATGGTCGAGGTGGATACGGACAAGGTGGAGCCGGTCAAGGCGGTGCAGGTGCTGC
TGCTGCGGCTGCTGCTGCCGGTGGTGCTGGTCAAGGTGGTCAAGGAGGCTATGGTCAAGG
TGGATACGGACAAGGTGGAGCTGGTCAAGGAGGTGCCGCTGCGGCTGCTGCTGCTGCTGC
AGGTGGTGCTGGTCAAGGTGGTTATGGCAGAGGAGGTGCTGGTCAAGGAGGTGCTGCTGC
TGCCGCAGCAGCTGCCGCAGGTGCAGGACAAGGAGGATATGGTGGACAAGGAGCCGGTCA
AGGAGGTGCAGGTGCAGCTGCCGCTGCTGCTGCTGCCGCTAGTGGTGGTGCTGGTCAAGG
TGGTCAAGGAGGCTATGGTCGAGGTGGATACGGACAAGGTGGAGCCGGTCAAGGCGGTGC
AGGTGCTGCTGCTGCGGCTGCTGCTGCCGGTGGTGCTGGTCAAGGTGGTCAAGGAGGCTA
TGGTCAAGGTGGATACGGACAAGGTGGAGCTGGTCAAGGAGGTGCCGCTGCGGCTGCTGC
TGCTGCTGCAGGTGGTGCTGGTCAAGGTGGTTATGGCAGAGGAGGTGCTGGTCAAGGAGG
TGCTGCTGCTGCCGCAGCAGCTGCCGCAGGTGCAGGACAAGGAGGATATGGTGGACAAGG
AGCCGGTCAAGGAGGTGCAGGTGCAGCTGCCGCTGCTGCTGCTGCCGGTGGTGCTGGTCA
AGGTGGTCAAGGAGGCTATGGTCGAGGTGGATACGGACAAGGTGGAGCCGGTCAAGGCGG
TGCAGGTGCTGCTGCTGCGGCTGCTGCTGCCGGTGGTGCTGGTCAAGGTGGTCAAGGAGG

CTATGGTCAAGGTGGATACGGACAAGGTGGAGCTGGTCAAGGAGGTGCCGCTGCGGCTGC
TGCTGCTGCTGCAGGTGGTGCTGGTCAAGGTGGTTATGGCAGAGGAGGTGCTGGTCAAGG
AGGTGCTGCTGCTGCCGCAGCAGCTGCCGCAGGTGCAGGACAAGGAGGATATGGTGGACA
AGGAGCCGGTCAAGGAGGTGCAGGTGCAGCTGCCGCTGCTGCTGCTGCCGCTAGCAGTGG
ACCTGGTCAAATTTATTATGGACCCCAATCTGTTGCTGCTCCAGCAGCAGCAGCAGCTTC
TGCTTTGGCAGCTCCAGCTACAAGCGCGAGAATTTCTTCACACGCCTCAGCTCTTCTTTC
AAATGGACCTACTAACCCTGCTTCTATTTCAAACGTTATTAGTAATGCTGTATCCCAAAT
TAGTTCCAGCAATCCAGGAGCGTCTGCGTGTGATGTTCTCGTTCAAGCTCTTCTTGAACT
TGTTACTGCTTTGCTCACCATTATTGGATCATCAAATATTGGCAGTGTTAATTATGATTC
TTCAGGCCAATATGCGCAAGTTGTTACTCAATCTGTTCAAAATGCATTCGCTGTCAGTTA
CGGAGCTGGCAGGGGATACGGACAAGGTGCAGGAAGTGCAGCTTCCTCTGTGTCATCTGC
TTCATCTCGCAGTTACGACTATTCTCGTCGTAACGTCCGCAAAAACTGTGGAATTCCTAG
AAGACAACTCGTTGTTAAATTCAGAGCACTGCCTTGTGTGAATTGCTAATTTTTAATATA
AAATAACCCTTGTTTCTTACTTCGTCCTGGATACATCTATGTTTTTTTTTCGTTAATAA
ATGAGAGCATTTAAGTTATTGTTTTTAATTACTTTTTTTAGAAAACAGATTTCGGATTT
TTTGTATGCATTTTATTTGAATGTACTAATATAATCAATTAATCAATGAATTCATTTATT
TAAGGGATAACAATAATCCATGAATTCACATGCACATTTAAAACAAAACTAAATTACAAT
AGGTTCATATAAAAACAACAAGTATGCCTTCTCAACTAAGAATACTATATTGTTTAAACC
GTAAAAAAAGTCATATTTCTGTATATCAAAACACATCTAATATTAAAAAAACAGTCAGCA
AGCACTTACAAGTGTGGGCTCGGACAGCAATTACCTGGTCTCAGGAGACCAATTGTTGTT
GTTAACTTGTTTATTGCAGCTTATAATGGTTACAAATAAAGCAATAGCATCACAAATTTC
ACAAATAAAGCATTTTTTTCACTGCATTCTAGTTGTGGTTTGTCCAAACTCATCAATGTA
TCTTAAGCTTGGCGCGCCGTACGCGTATCGATAAGCTTTAAGATACATTGATGAGTTTGG
ACAAACCACAACTAGAATGCAGTGAAAAAAATGCTTTATTTGTGAAATTTGTGATGCTAT
TGCTTTATTTGTAACCATTATAAGCTGCAATAAACAAGTTAACAACAACAATTGCATTCA
TTTTATGTTTCAGGTTCAGGGGGAGGTGTGGGAGGTTTTTTAAAGCAAGTAAAACCTCTA
CAAATGTGGTATGGCTGATTATGATCTAGAGTCGCGGCCGCTACAGGAACAGGTGGTGGC
GGCCCTCGGTGCGCTCGTACTGCTCCACGATGGTGTAGTCCTCGTTGTGGGAGGTGATGT
CCAGCTTGGAGTCCACGTAGTAGTAGCCGGGCAGCTGCACGGGCTTCTTGGCCATGTAGA
TGGACTTGAACTCCACCAGGTAGTGGCCGCCGTCCTTCAGCTTCAGGGCCTTGTGGATCT
CGCCCTTCAGCACGCCGTCGCGGGGGTACAGGCGCTCGGTGGAGGCCTCCCAGCCCATGG
TCTTCTTCTGCATTACGGGGCCGTCGGAGGGGAAGTTCACGCCGATGAACTTCACCTTGT
AGATGAAGCAGCCGTCCTGCAGGGAGGAGTCTTGGGTCACGGTCACCACGCCGCCGTCCT
CGAAGTTCATCACGCGCTCCCACTTGAAGCCCTCGGGGAAGGACAGCTTCTTGTAGTCGG
GGATGTCGGCGGGGTGCTTCACGTACACCTTGGAGCCGTACTGGAACTGGGGGGACAGGA
TGTCCCAGGCGAAGGGCAGGGGGCCGCCCTTGGTCACCTTCAGCTTCACGGTGTTGTGGC
CCTCGTAGGGGCGGCCCTCGCCCTCGCCCTCGATCTCGAACTCGTGGCCGTTCACGGTGC
CCTCCATGCGCACCTTGAAGCGCATGAACTCCTTGATGACGTTCTTGGAGGAGCGCACCA
TGGTGGCGACCGGTGGATCCCGGGCCCGCGGTACCGTCGACTCTAGCGGTACCCCGATTG
TTTAGCTTGTTCAGCTGCGCTTGTTTATTTGCTTAGCTTTCGCTTAGCGACGTGTTCACT
TTGCTTGTTTGAATTGAATTGTCGCTCCGTAGACGAAGCGCCTCTATTTATACTCCGGCG
GTCGAGGGTTCGAAATCGATAAGCTTGGATCCTAATTGAATTAGCTCTAATTGAATTAGT
CTCTAATTGAATTAGATCCCCGGGC

Supplementary data S2 The important component sequence of pBac[3×P3-DsRed]-MaSp2×24 expression vector.

>The important component sequence of pBac[3×P3-DsRed]-MaSp2×24 expression vector

Fib-H promoter

Fib-H signal peptide

MaSp2 repetitive unit×24

CTD of MaSp2

CTD of Fib-H -Poly A of Fib-H

The partial SV40

SV40 poly A-DsRed- 3×P3 promoter

The key component sequences were marked with the corresponding colour.

CTCGAGCAGCTCTAACACTAGGCTAACTCAGGCTTAGTAGCCTGGTCCTAGTGTTAGATT
TGAAGTCGTCTAATGCAAAGATTATTGGATCTGATGGATCCGTAAGGACGTGTCTAGAGC
GTCGACGGTGACTAGCTCCTGCGTGATCAGGAAAAATGTGGAAAGCTTAACGATTTTGTC
ACATTTTACTTATCACAACTTGTTTTTATAATAATTCGCTTAAATGAGCAGCTATTACTT
AATCTCGTAGTGGTTTTTGACAAAATCAGCTTCTTTAGAACTAAAATATCATTTTTTTCG
TAATTTTTTTAATGAAAAATGCTCTAGTGTTATACCTTTCCAAAATCACCATTAATTAGG
TAGTGTTTAAGCTTGTTGTACAAAACTGCCACACGCATTTTTTTCTCCACTGTAGGTTGT
AGTTACGCGAAAACAAAATCGTTCTGTGAAAATTCAAACAAAAATATTTTTTCGTAAAAA
CACTTATCAATGAGTAAAGTAACAATTCATGAATAATTTCATGTAAAAAAAAAATACTAG
AAAAGGAATTTTTCATTACGAGATGCTTAAAAATCTGTTTCAAGGTAGAGATTTTTCGAT
ATTTCGGAAAATTTTGTAAAACTGTAAATCCGTAAAATTTTGCTAAACATATATTGTGTT
GTTTTGGTAAGTATTGACCCAAGCTATCACCTCCTGCAGTATGTCGTGCTAATTACTGGA
CACATTGTATAACAGTTCCACTGTATTGACAATAATAAAACCTCTTCATTGACTTGAGAA
TGTCTGGACAGATTTGGCTTTGTATTTTTGATTTACAAATGTTTTTTTGGTGATTTACCC
ATCCAAGGCATTCTCCAGGATGGTTGTGGCATCACGCCGATTGGCAAACAAAAACTAAAA
TGAAACTAAAAAGAAACAGTTTCCGCTGTCCCGTTCCTCTAGTGGGAGAAAGCATGAAGT
AAGTTCTTTAAATATTACAAAAAAATTGAACGATATTATAAAATTCTTTAAAATATTAAA
AGTAAGAACAATAAGATCAATTAAATCATAATTAATCACATTGTTCATGATCACAATTTA
ATTTACTTCATACGTTGTATTGTTATGTTAAATAAAAAGATTAATTTCTATGTAATTGTA
TCTGTACAATACAATGTGTAGATGTTTATTCTATCGAAAGTAAATACGTCAAAACTCGAA
AATTTTCAGTATAAAAAGGTTCAACTTTTTCAAATCAGCATCAGTTCGGTTCCAACTCTC
AAGCCATGGATGAGAGTCAAAACCTTTGTGATCTTGTGCTGCGCTCTGCAGTATGTCGCT
TATACAAATGCAACTAGTGGTGGTGCTGGTCCTGGAAGACAACAGGCTTATGGTCCAGGA
GGTTCTGGTGCTGCTGCGGCTGCTGCTGCAGGTGGTGCTGGTCCTGGAAGACAACAAGC
CTATGGTCCAGGAGGTTCAGGTGCTGCTGCGGCTGCTGCTGCAGGTGGTCCAGGTTACGG
TGGACAACAAGGATACGGACCAGGTGGTGCTGGAGCTGCTGCAGCAGCTGCCGCAGGTGG
TGCTGGTCCTGGAAGACAACAAGCATATGGTCCAGGAGGTTCTGGTGCTGCTGCGGCTGC
TGCTGCAGGTGGTGCTGGTCCTGGAAGACAACAAGCCTATGGTCCAGGAGGTTCAGGTGC
TGCTGCGGCTGCTGCTGCAGGTGGTCCAGGTTACGGTGGACAACAAGGATACGGACCAGG

TGGTGCTGGAGCTGCTGCAGCAGCTGCCGCAGGTGGTGCTGGTCCTGGAAGACAACAAGC
ATATGGTCCAGGAGGTTCTGGTGCTGCTGCGGCTGCTGCTGCAGGTGGTGCTGGTCCTGG
AAGACAACAAGCCTATGGTCCAGGAGGTTCAGGTGCTGCTGCGGCTGCTGCTGCAGGTGG
TCCAGGTTACGGTGGACAACAAGGATACGGACCAGGTGGTGCTGGAGCTGCTGCAGCAGC
TGCCGCAGCTAGTGGTGGTGCTGGTCCTGGAAGACAACAGGCTTATGGTCCAGGAGGTTC
TGGTGCTGCTGCGGCTGCTGCTGCAGGTGGTGCTGGTCCTGGAAGACAACAAGCCTATGG
TCCAGGAGGTTCAGGTGCTGCTGCGGCTGCTGCTGCAGGTGGTCCAGGTTACGGTGGACA
ACAAGGATACGGACCAGGTGGTGCTGGAGCTGCTGCAGCAGCTGCCGCAGGTGGTGCTGG
TCCTGGAAGACAACAAGCATATGGTCCAGGAGGTTCTGGTGCTGCTGCGGCTGCTGCTGC
AGGTGGTGCTGGTCCTGGAAGACAACAAGCCTATGGTCCAGGAGGTTCAGGTGCTGCTGC
GGCTGCTGCTGCAGGTGGTCCAGGTTACGGTGGACAACAAGGATACGGACCAGGTGGTGC
TGGAGCTGCTGCAGCAGCTGCCGCAGGTGGTGCTGGTCCTGGAAGACAACAAGCATATGG
TCCAGGAGGTTCTGGTGCTGCTGCGGCTGCTGCTGCAGGTGGTGCTGGTCCTGGAAGACA
ACAAGCCTATGGTCCAGGAGGTTCAGGTGCTGCTGCGGCTGCTGCTGCAGGTGGTCCAGG
TTACGGTGGACAACAAGGATACGGACCAGGTGGTGCTGGAGCTGCTGCAGCAGCTGCCGC
AGCTAGTGGTGGTGCTGGTCCTGGAAGACAACAGGCTTATGGTCCAGGAGGTTCTGGTGC
TGCTGCGGCTGCTGCTGCAGGTGGTGCTGGTCCTGGAAGACAACAAGCCTATGGTCCAGG
AGGTTCAGGTGCTGCTGCGGCTGCTGCTGCAGGTGGTCCAGGTTACGGTGGACAACAAGG
ATACGGACCAGGTGGTGCTGGAGCTGCTGCAGCAGCTGCCGCAGGTGGTGCTGGTCCTGG
AAGACAACAAGCATATGGTCCAGGAGGTTCTGGTGCTGCTGCGGCTGCTGCTGCAGGTGG
TGCTGGTCCTGGAAGACAACAAGCCTATGGTCCAGGAGGTTCAGGTGCTGCTGCGGCTGC
TGCTGCAGGTGGTCCAGGTTACGGTGGACAACAAGGATACGGACCAGGTGGTGCTGGAGC
TGCTGCAGCAGCTGCCGCAGGTGGTGCTGGTCCTGGAAGACAACAAGCATATGGTCCAGG
AGGTTCTGGTGCTGCTGCGGCTGCTGCTGCAGGTGGTGCTGGTCCTGGAAGACAACAAGC
CTATGGTCCAGGAGGTTCAGGTGCTGCTGCGGCTGCTGCTGCAGGTGGTCCAGGTTACGG
TGGACAACAAGGATACGGACCAGGTGGTGCTGGAGCTGCTGCAGCAGCTGCCGCAGCTAG
TGGTGGTGCTGGTCCTGGAAGACAACAGGCTTATGGTCCAGGAGGTTCTGGTGCTGCTGC
GGCTGCTGCTGCAGGTGGTGCTGGTCCTGGAAGACAACAAGCCTATGGTCCAGGAGGTTC
AGGTGCTGCTGCGGCTGCTGCTGCAGGTGGTCCAGGTTACGGTGGACAACAAGGATACGG
ACCAGGTGGTGCTGGAGCTGCTGCAGCAGCTGCCGCAGGTGGTGCTGGTCCTGGAAGACA
ACAAGCATATGGTCCAGGAGGTTCTGGTGCTGCTGCGGCTGCTGCTGCAGGTGGTGCTGG
TCCTGGAAGACAACAAGCCTATGGTCCAGGAGGTTCAGGTGCTGCTGCGGCTGCTGCTGC
AGGTGGTCCAGGTTACGGTGGACAACAAGGATACGGACCAGGTGGTGCTGGAGCTGCTGC
AGCAGCTGCCGCAGGTGGTGCTGGTCCTGGAAGACAACAAGCATATGGTCCAGGAGGTTC
TGGTGCTGCTGCGGCTGCTGCTGCAGGTGGTGCTGGTCCTGGAAGACAACAAGCCTATGG
TCCAGGAGGTTCAGGTGCTGCTGCGGCTGCTGCTGCAGGTGGTCCAGGTTACGGTGGACA
ACAAGGATACGGACCAGGTGGTGCTGGAGCTGCTGCAGCAGCTGCCGCAGCTAGTGGTGG
TGCTGGTCCTGGAAGACAACAGGCTTATGGTCCAGGAGGTTCTGGTGCTGCTGCGGCTGC
TGCTGCAGGTGGTGCTGGTCCTGGAAGACAACAAGCCTATGGTCCAGGAGGTTCAGGTGC
TGCTGCGGCTGCTGCTGCAGGTGGTCCAGGTTACGGTGGACAACAAGGATACGGACCAGG
TGGTGCTGGAGCTGCTGCAGCAGCTGCCGCAGGTGGTGCTGGTCCTGGAAGACAACAAGC
ATATGGTCCAGGAGGTTCTGGTGCTGCTGCGGCTGCTGCTGCAGGTGGTGCTGGTCCTGG
AAGACAACAAGCCTATGGTCCAGGAGGTTCAGGTGCTGCTGCGGCTGCTGCTGCAGGTGG
TCCAGGTTACGGTGGACAACAAGGATACGGACCAGGTGGTGCTGGAGCTGCTGCAGCAGC

TGCCGCAGGTGGTGCTGGTCCTGGAAGACAACAAGCATATGGTCCAGGAGGTTCTGGTGC
TGCTGCGGCTGCTGCTGCAGGTGGTGCTGGTCCTGGAAGACAACAAGCCTATGGTCCAGG
AGGTTCAGGTGCTGCTGCGGCTGCTGCTGCAGGTGGTCCAGGTTACGGTGGACAACAAGG
ATACGGACCAGGTGGTGCTGGAGCTGCTGCAGCAGCTGCCGCAGCTAGTGGTGGTGCTGG
TCCTGGAAGACAACAGGCTTATGGTCCAGGAGGTTCTGGTGCTGCTGCGGCTGCTGCTGC
AGGTGGTGCTGGTCCTGGAAGACAACAAGCCTATGGTCCAGGAGGTTCAGGTGCTGCTGC
GGCTGCTGCTGCAGGTGGTCCAGGTTACGGTGGACAACAAGGATACGGACCAGGTGGTGC
TGGAGCTGCTGCAGCAGCTGCCGCAGGTGGTGCTGGTCCTGGAAGACAACAAGCATATGG
TCCAGGAGGTTCTGGTGCTGCTGCGGCTGCTGCTGCAGGTGGTGCTGGTCCTGGAAGACA
ACAAGCCTATGGTCCAGGAGGTTCAGGTGCTGCTGCGGCTGCTGCTGCAGGTGGTCCAGG
TTACGGTGGACAACAAGGATACGGACCAGGTGGTGCTGGAGCTGCTGCAGCAGCTGCCGC
AGGTGGTGCTGGTCCTGGAAGACAACAAGCATATGGTCCAGGAGGTTCTGGTGCTGCTGC
GGCTGCTGCTGCAGGTGGTGCTGGTCCTGGAAGACAACAAGCCTATGGTCCAGGAGGTTC
AGGTGCTGCTGCGGCTGCTGCTGCAGGTGGTCCAGGTTACGGTGGACAACAAGGATACGG
ACCAGGTGGTGCTGGAGCTGCTGCAGCAGCTGCCGCAGCTAGTGGTGGTGCTGGTCCTGG
AAGACAACAGGCTTATGGTCCAGGAGGTTCTGGTGCTGCTGCGGCTGCTGCTGCAGGTGG
TGCTGGTCCTGGAAGACAACAAGCCTATGGTCCAGGAGGTTCAGGTGCTGCTGCGGCTGC
TGCTGCAGGTGGTCCAGGTTACGGTGGACAACAAGGATACGGACCAGGTGGTGCTGGAGC
TGCTGCAGCAGCTGCCGCAGGTGGTGCTGGTCCTGGAAGACAACAAGCATATGGTCCAGG
AGGTTCTGGTGCTGCTGCGGCTGCTGCTGCAGGTGGTGCTGGTCCTGGAAGACAACAAGC
CTATGGTCCAGGAGGTTCAGGTGCTGCTGCGGCTGCTGCTGCAGGTGGTCCAGGTTACGG
TGGACAACAAGGATACGGACCAGGTGGTGCTGGAGCTGCTGCAGCAGCTGCCGCAGGTGG
TGCTGGTCCTGGAAGACAACAAGCATATGGTCCAGGAGGTTCTGGTGCTGCTGCGGCTGC
TGCTGCAGGTGGTGCTGGTCCTGGAAGACAACAAGCCTATGGTCCAGGAGGTTCAGGTGC
TGCTGCGGCTGCTGCTGCAGGTGGTCCAGGTTACGGTGGACAACAAGGATACGGACCAGG
TGGTGCTGGAGCTGCTGCAGCAGCTGCCGCAGCTAGTGGTGGTGCTGGTCCTGGAAGACA
ACAGGCTTATGGTCCAGGAGGTTCTGGTGCTGCTGCGGCTGCTGCTGCAGGTGGTGCTGG
TCCTGGAAGACAACAAGCCTATGGTCCAGGAGGTTCAGGTGCTGCTGCGGCTGCTGCTGC
AGGTGGTCCAGGTTACGGTGGACAACAAGGATACGGACCAGGTGGTGCTGGAGCTGCTGC
AGCAGCTGCCGCAGGTGGTGCTGGTCCTGGAAGACAACAAGCATATGGTCCAGGAGGTTC
TGGTGCTGCTGCGGCTGCTGCTGCAGGTGGTGCTGGTCCTGGAAGACAACAAGCCTATGG
TCCAGGAGGTTCAGGTGCTGCTGCGGCTGCTGCTGCAGGTGGTCCAGGTTACGGTGGACA
ACAAGGATACGGACCAGGTGGTGCTGGAGCTGCTGCAGCAGCTGCCGCAGGTGGTGCTGG
TCCTGGAAGACAACAAGCATATGGTCCAGGAGGTTCTGGTGCTGCTGCGGCTGCTGCTGC
AGGTGGTGCTGGTCCTGGAAGACAACAAGCCTATGGTCCAGGAGGTTCAGGTGCTGCTGC
GGCTGCTGCTGCAGGTGGTCCAGGTTACGGTGGACAACAAGGATACGGACCAGGTGGTGC
TGGAGCTGCTGCAGCAGCTGCCGCAGCTAGCAGTGGATCAGGTGGATATGGTCCTTCACA
ATATGTTCCTAGCTCTGTTGCTTCTAGTGCTGCATCAGCAGCCTCAGCTTTATCTTCACC
GACAACGCATGCTAGAATTTCTTCCCATGCATCAACTCTATTATCAAGTGGGCCAACTAA
TGCGGCAGCTCTTTCTAATGTCATTAGTAATGCCGTTTCCCAAGTCAGTGCAAGTAATCC
AGGATCTTCCTCTTGTGATGTCCTTGTTCAAGCACTTCTTGAAATAATTACTGCATTAAT
TAGTATACTAGATTCCTCTAGTGTTGGACAAGTTAATTACGGTTCTTCAGGACAGTATGC
ACAAATTGTAGGGCAGTCTATGCAACAGGCTATGGGGGTCAGTTACGGAGCTGGCAGGGG
ATACGGACAAGGTGCAGGAAGTGCAGCTTCCTCTGTGTCATCTGCTTCATCTCGCAGTTA

CGACTATTCTCGTCGTAACGTCCGCAAAAACTGTGGAATTCCTAGAAGACAACTCGTTGT
TAAATTCAGAGCACTGCCTTGTGTGAATTGCTAATTTTTAATATAAAATAACCCTTGTTT
CTTACTTCGTCCTGGATACATCTATGTTTTTTTTTTCGTTAATAAATGAGAGCATTTAAG
TTATTGTTTTTAATTACTTTTTTTTAGAAAACAGATTTCGGATTTTTTGTATGCATTTTA
TTTGAATGTACTAATATAATCAATTAATCAATGAATTCATTTATTTAAGGGATAACAATA
ATCCATGAATTCACATGCACATTTAAAACAAAACTAAATTACAATAGGTTCATATAAAAA
CAACAAGTATGCCTTCTCAACTAAGAATACTATATTGTTTAAACCGTAAAAAAAGTCATA
TTTCTGTATATCAAAACACATCTAATATTAAAAAAACAGTCAGCAAGCACTTACAAGTGT
GGGCTCGGACAGCAATTACCTGGTCTCAGGAGACCAATTGTTGTTGTTAACTTGTTTATT
GCAGCTTATAATGGTTACAAATAAAGCAATAGCATCACAAATTTCACAAATAAAGCATTT
TTTTCACTGCATTCTAGTTGTGGTTTGTCCAAACTCATCAATGTATCTTAAGCTTGGCGC
GCCGTACGCGTATCGATAAGCTTTAAGATACATTGATGAGTTTGGACAAACCACAACTAG
AATGCAGTGAAAAAAATGCTTTATTTGTGAAATTTGTGATGCTATTGCTTTATTTGTAAC
CATTATAAGCTGCAATAAACAAGTTAACAACAACAATTGCATTCATTTTATGTTTCAGGT
TCAGGGGGAGGTGTGGGAGGTTTTTTAAAGCAAGTAAAACCTCTACAAATGTGGTATGGC
TGATTATGATCTAGAGTCGCGGCCGCTACAGGAACAGGTGGTGGCGGCCCTCGGTGCGCT
CGTACTGCTCCACGATGGTGTAGTCCTCGTTGTGGGAGGTGATGTCCAGCTTGGAGTCCA
CGTAGTAGTAGCCGGGCAGCTGCACGGGCTTCTTGGCCATGTAGATGGACTTGAACTCCA
CCAGGTAGTGGCCGCCGTCCTTCAGCTTCAGGGCCTTGTGGATCTCGCCCTTCAGCACGC
CGTCGCGGGGGTACAGGCGCTCGGTGGAGGCCTCCCAGCCCATGGTCTTCTTCTGCATTA
CGGGGCCGTCGGAGGGGAAGTTCACGCCGATGAACTTCACCTTGTAGATGAAGCAGCCGT
CCTGCAGGGAGGAGTCTTGGGTCACGGTCACCACGCCGCCGTCCTCGAAGTTCATCACGC
GCTCCCACTTGAAGCCCTCGGGGAAGGACAGCTTCTTGTAGTCGGGGATGTCGGCGGGGT
GCTTCACGTACACCTTGGAGCCGTACTGGAACTGGGGGGACAGGATGTCCCAGGCGAAGG
GCAGGGGGCCGCCCTTGGTCACCTTCAGCTTCACGGTGTTGTGGCCCTCGTAGGGGCGGC
CCTCGCCCTCGCCCTCGATCTCGAACTCGTGGCCGTTCACGGTGCCCTCCATGCGCACCT
TGAAGCGCATGAACTCCTTGATGACGTTCTTGGAGGAGCGCACCATGGTGGCGACCGGTG
GATCCCGGGCCCGCGGTACCGTCGACTCTAGCGGTACCCCGATTGTTTAGCTTGTTCAGC
TGCGCTTGTTTATTTGCTTAGCTTTCGCTTAGCGACGTGTTCACTTTGCTTGTTTGAATT
GAATTGTCGCTCCGTAGACGAAGCGCCTCTATTTATACTCCGGCGGTCGAGGGTTCGAAA
TCGATAAGCTTGGATCCTAATTGAATTAGCTCTAATTGAATTAGTCTCTAATTGAATTAG
ATCCCCGGGC

The unprocessed original scans for all of the blots in all Figures:

The full-length SDS-PAGE gels and blots of Figure 4

(a)

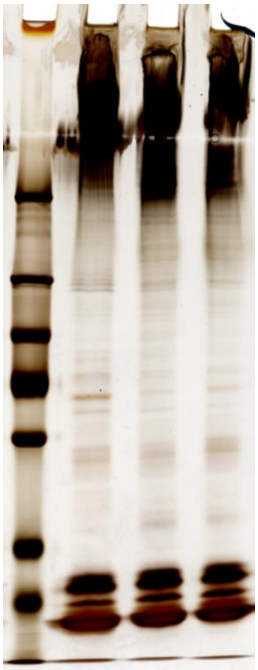

(b)

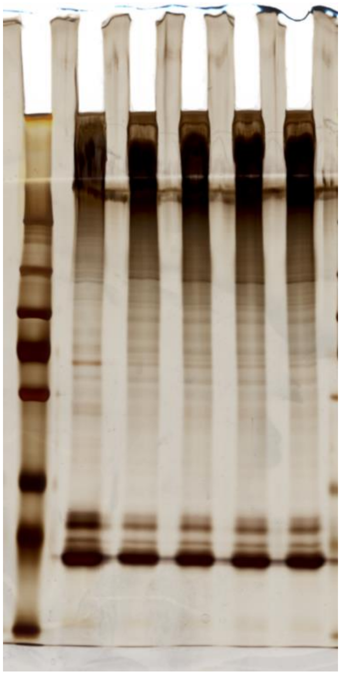

(c)

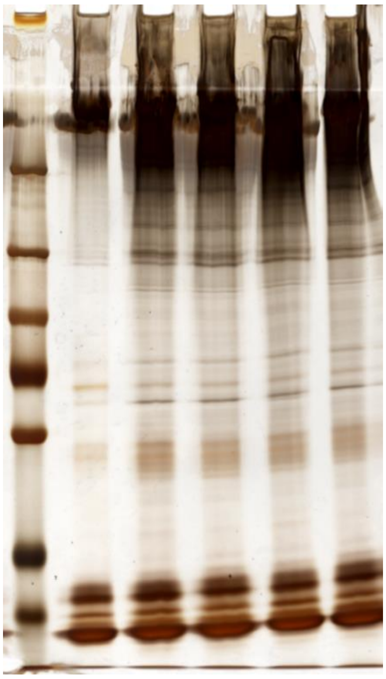

(d)

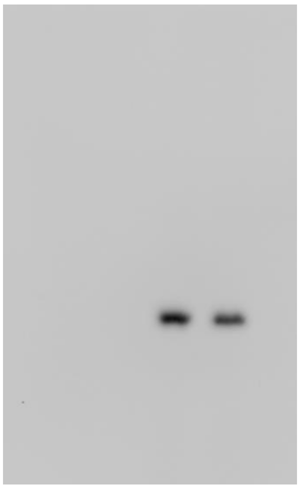

(e)

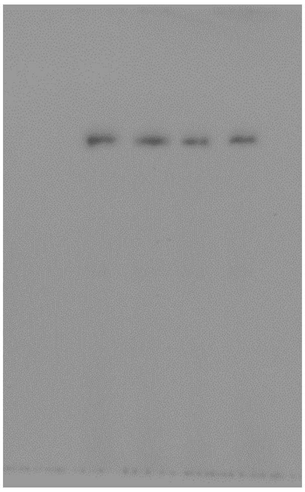

(f)

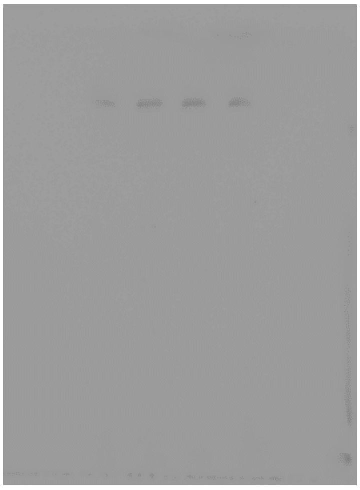

(a) The full-length SDS-PAGE gel of Figure 4b; **(b)** the full-length SDS-PAGE gel of Figure 4b′ ; **(c)** the full-length gels of Figure 4b″ ; **(d)** the full-length blots of Figure 4c; **(e)** the full-length blots of Figure 4c′ ; **(f)** the full-length blots of Figure 4c″ .

The full-length SDS-PAGE gel and blots of Supplementary Figure S6

(a)

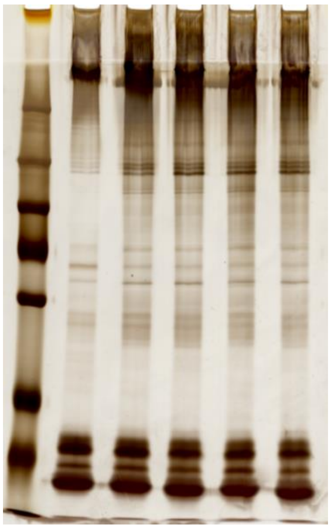

(b)

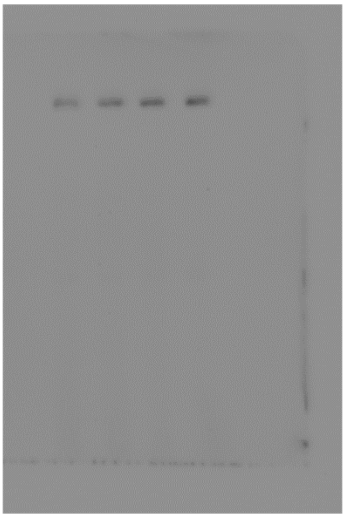

(a) The full-length SDS-PAGE gels of Supplementary Figure S6b; **(b)** the full-length blots of Supplementary Figure S6c
